# Group conditions for entrepreneurial visions: role confidence, hierarchical congruences, and the imagining of future in entrepreneurial groups

**DOI:** 10.1007/s11187-021-00566-6

**Published:** 2021-11-30

**Authors:** Isabell Stamm, Marie Gutzeit

**Affiliations:** Department for Sociology, Technische Universität Berlin, Berlin, Germany

**Keywords:** Entrepreneurship, Economic sociology, Future, Groups, Roles, Collective agency, D8, L26

## Abstract

An essential part of entrepreneurial activity is the drafting and narrating of an entrepreneurial vision. This study is premised on the observation that entrepreneurial groups form an interaction arena for the practice of imagining the future and asks how the content of entrepreneurial visions is shaped by the conditions of the group. Taking an entrepreneurship-as-practice lens, which we enrich with sociological theory on the future (Beckert) and small groups (Fine), we engage in an in-depth case study of 12 entrepreneurial groups. We show how the content of entrepreneurial visions is configured by four elements (i.e., fictional expectation for the business or the group; future orientation that is continuing or divergent) and name two group conditions (i.e., role confidence and hierarchical congruence) that direct their configuration. We propose that lacking role confidence can impede thinking about the future of a business and that narrative hierarchies that challenge structural hierarchies can open a window for divergent future orientation. This study contributes to a novel theoretical understanding of where entrepreneurial visions come from by emphasizing politics of expectations within groups and calling to consider group conditions as a relevant context for entrepreneurial visions.

**Supplementary Information:**

The online version contains supplementary material available at 10.1007/s11187-021-00566-6.

## Abstract (plain language summary)

Entrepreneurs make futures! To do so, they have to be confident in their role within an entrepreneurial group or they will be caught up in thinking about how the group should work together rather than drafting a future for the business. This is a key finding of the study put forward by Stamm and Gutzeit on the practice of imagining the future. Family members, friends, or former colleagues who jointly engage in an entrepreneurial venture form such a group, with its own history, structure, and culture. The authors’ qualitative study, based on group interviews, reveals that these group conditions influence the content of entrepreneurial visions. For entrepreneurship research, this study implies that an entrepreneurial vision is a product of group political processes rather than individual mental images. This study further implies that entrepreneurial groups and their advisors should reflect on group conditions before engaging in the drafting of an entrepreneurial vision.

**Supplementary Information:**

The online version contains supplementary material available at 10.1007/s11187-021-00566-6.

## Introduction

Entrepreneurial visions have been shown to provide entrepreneurial action with direction and to contribute to entrepreneurial success. Entrepreneurs act under the condition of uncertainty (Alvarez & Barney, 2007; McKelvie et al., 2011; McMullen & Shepherd, 2006; Packard et al., 2017; Wood et al., 2021). In this situation, they formulate an individual entrepreneurial vision for their ventures, which Filion has defined as “an image projected into the future of the market space to be occupied by the products and the type of organization needed to achieve this” (Filion, 1991, pp. 109–110). Gartner has referred to these entrepreneurial visions as “elaborate fictions of a proposed possible future state” (Gartner et al., 1992, p. 17), which reduce uncertainty and make it possible to “act as if’ these fictions were real.” An entrepreneurial vision helps to identify opportunity (Filion, 2004; Kammerlander et al., 2016) and impact organizational performance (Jaskiewicz et al., 2015; Kantabutra, 2009), as well as continuity and growth (Baum et al., 1998; O'Connell et al., 2011; Ruvio et al., 2010; Barbera et. al, 2018).

Recently, researchers have raised the question of where such entrepreneurial visions actually come from (Gartner, 2016; Preller et al., 2020; Wood et al., 2021). This puzzle pertains to both the content of the image of the future as well as the process of imagining the future. Commonly, an entrepreneurial vision is argued to be personal (Preller et al., 2020) or internal (Wood et al., 2021), and hence, the content of the image of the future needs to be treated as the property of individuals (e.g., Brush, 2008; Miller & Le Breton-Miller, 2017; O'Connell et al., 2011). From this perspective, imagining the future appears as a cognitive process during which individuals draft internal narratives based on the individual’s knowledge frame (Haynie et al., 2010; Wood et al., 2021). This approach strongly ties an entrepreneurial vision to the entrepreneurial individual.

Such a personal view of entrepreneurial visions, however, only provides a partial understanding of where these visions come from. When engaging in venturing activity, entrepreneurs are prompted at some stage to share their entrepreneurial vision with others (e.g., Aldrich & Fiol, 1994; Garud et al., 2014; Lounsbury & Glynn, 2001). Wood et al. (2021) consider this to be “externalization”—through the verbalization of what were previously internal narratives that entrepreneurs crafted for themselves; the image of the future for the venture now enters social interaction.

Preller et al. (2020) point out that entrepreneurial teams are an important interaction arena in which a personal entrepreneurial vision is shared. They thus follow a growing stream of research within the field of entrepreneurship (Klotz et al., 2014), which points towards teams as important social units collectively acting entrepreneurially and shaping the venture (Ruef, 2010). More specifically, Preller et al. (2020) critique previous research for assuming that there is only one image of the future, which is defined by the lead entrepreneur (e.g., Ashford et al., 2018) or resembles agreement within the founding team (Knockaert et al., 2011). Instead, they argue, in line with Ensley et al. (2003), that an individual’s image of the venture’s future enters a team-level interaction arena and needs to be aligned at least to some extent with others. Each individual can hold a different entrepreneurial vision and the overlapping content of these future images sets the venture on different trajectories for opportunity development (Preller et al., 2020). Wood et al. (2021) agree that there can be significant agonizing over these entrepreneurial visions.

These insights indicate that the content of entrepreneurial visions is not static (Filion, 2004), but continues to form and morph during social interaction. Narrative approaches to entrepreneurship even argue that an entrepreneurial vision is socially constructed during such social interaction (e.g., Aldrich & Fiol, 1994; Garud et al., 2014; Lounsbury & Glynn, 2001). These social interactions, however, are not limited to those holding a formal position in the newly emergent organization as the concept of entrepreneurial teams implies (Harper, 2008). Rather, the small number of individuals emotionally, socially, or financially engaged in the venturing activities (independent of their formal position) may weigh in and be an important source for the shaping of an entrepreneurial vision. Needed is thus a more inclusive small-group perspective on imagining future, and in the following, we utilize the term entrepreneurial group (Ruef, 2010) to make this point. In entrepreneurial groups, as a specific and relevant interaction arena, images of the venture’s future are filtered and negotiated, and the conditions of the group may constrain innovative potential and visions for the venture. Yet to date, we do not have a theory that adequately describes the link between entrepreneurial groups and the content of entrepreneurial visions in general, and the effect of group conditions on images of the future in particular. Such a theory, however, is essential in order to develop a holistic understanding of where entrepreneurial visions come from.

We devote our study to developing grounded theory on entrepreneurial groups as interaction arenas for imagining the future. To do so, we connect to an “entrepreneurship as practice” perspective (Gartner et al., 2016; Thompson et al., 2020) and frame imagining the future as an entrepreneurial practice, which is independent of the stage and context of the venture. We then turn to Beckert’s sociological theory of “imagined futures” (Beckert, 2013, 2016, 2021) and to Fines’ sociological theory of “group action and culture” (Fine, 2012) to emphasize how the practice of imagining the future within small groups is political in the sense of a discursive negotiation and competition that shapes the content of entrepreneurial visions. As an exemplary context, we turn to ventures in German crafts, where entrepreneurial groups are under pressure to draft entrepreneurial visions in the light of massive sectoral changes, including a skilled worker shortage, increased competition, digitalization, and a looming succession wave. We probe how entrepreneurial groups generate and modify their images of the future. We utilize a methodological approach that fulfills the demands of a practice perspective by capturing interactions and group practices in situ (Johannisson, 2011); we conducted a comparative study of 12 cases, featuring group interviews complemented by additional expert interviews and document research. We analyzed the data material using a Gioia-inspired approach to grounded theory (Gioia et al., 2012), and integrated documentary method (Bohnsack, 2010) and visualizing techniques, which allowed us to analyze the content of the entrepreneurial visions, the discursive and group dynamics, and their interlinking.

Contributing to entrepreneurship as a science of imagining (Gartner, 2007), we present a model that shows how the content of the entrepreneurial vision and its imagined potential for change is moderated at large by two group conditions. We find that entrepreneurial visions encompass not only images for the future of the business, but also for the group. We firstly propose that the group condition of role confidence affects the ability to focus on the future of the business rather than being preoccupied with the future of the group. We secondly propose that the group condition of incongruence between structural and narrative hierarchy opens a window to formulate change-oriented entrepreneurial visions. In other words, we unveil essential group conditions that need to be met in order for entrepreneurial groups to draft an entrepreneurial vision and for this future to diverge from established paths. By providing a more complex approach to both imagining the future and entrepreneurial groups, this study makes leeway in understanding where entrepreneurial visions come from. Knowledge of how an entrepreneurial vision is shaped sheds light on mediating factors of entrepreneurial activity and ultimately contributes to learning about diverging entrepreneurial pathways in terms of innovativeness, performance, and growth.

## Theoretical framework

### Imagining the future as practice

Entrepreneurship scholars increasingly turn to the emerging “entrepreneurship as practice” perspective (EaP) as a way to approach the collective action of venturing. This perspective neither reduces properties of practices to individual behaviors, cognitions, or motivations, nor overemphasizes the structuring power of contextual factors.

EaP calls for scholars to approach venturing from entrepreneurial practices, defined as bundles of activities targeted towards “getting things done” (Gartner et al., 2016; Johannisson, 2011). Previous studies have examined a variety of entrepreneurial practices, including entrepreneurial marketing (Gross et al., 2014), new venture legitimation (Clercq & Voronov, 2009), networking (Johannisson, 2011), or pitching (Chalmers & Shaw, 2017). In venturing, multiple practices interconnect to form a continuous unfolding and genuinely collective process (Johannisson, 2011; Steyaert, 2007).

Following the EaP perspective, we frame imagining the future as an important venturing practice that consists of drafting and narrating an entrepreneurial vision, i.e., images of the venture’s future. As such, this practice binds entrepreneurial activities that have been elaborated upon in previous research, such as entrepreneurial imagination (Keating & McLoughlin, 2010; Kier & McMullen, 2018; Miller & Le Breton-Miller, 2017) and entrepreneurial storytelling (Downing, 2005; Johansson, 2004). The in-practice constructed and reconstructed entrepreneurial vision guides entrepreneurial activity and motivates others to engage with the venture (Gartner et al., 1992; Garud et al., 2014). It is important to note that entrepreneurs as agents arise in the process of venturing, and that their bodily and mental activities are elements of these collective entrepreneurial practices and not qualities of entrepreneurs themselves (Steyaert, 2007).

Further, imagining the future is not a singular act reserved for the early stages of a venture, but rather an ongoing and essential element of venturing (Bird, 1988; Filion, 2004). Additionally, imagining the future is not just tied to entrepreneurs with innovation and high-growth aspirations, but is an essential element for all everyday entrepreneurial ventures—in the same way, Welter et al. (2016) refer to the heterogeneity of entrepreneurship. New technologies, changing lifestyles, new competitors, or economic crises, among other things, may increase uncertainty for entrepreneurs and may make the development of once anticipated ventures ambiguous. In such situations, the (re)drafting of an entrepreneurial vision reduces uncertainty (Garud et al., 2014) and motivates entrepreneurial activity by offering plausible explanations for current and future developments (Gartner et al., 1992).

Framing entrepreneurial visions as narratives of images of the future constructed in the practice of imagining the future expands a personal view of entrepreneurial visions (e.g., Preller et al., 2020; Wood et al., 2021). It situates the personal mental images within the discursive negotiation of entrepreneurial visions, emphasizes the dynamic character of the content of entrepreneurial visions, and thus creates a conceptual touchstone to study where entrepreneurial visions come from.

### The content of entrepreneurial visions

From an EaP perspective, the content of an entrepreneurial vision is socially constructed in the practice of imagining the future. This content is thus not simply a product of an individual’s mind or even a group discussion; rather, EaP scholars demand that we include the broader contextual world to understand how an entrepreneurial vision forms in this practice (Gartner et al., 2016; Gross et al., 2014). In this regard, Beckert’s sociological theory of imagined futures (Beckert, 2016, 2017a, 2017b, 2013) is particularly informative.

Beckert starts from the assumption that all economic actions occur under the prerequisite of fundamental uncertainty (Beckert, 2016), as does research on entrepreneurial action (Alvarez & Barney, 2007; McKelvie et al., 2011). A range of “possible futures” exists (Uprichard, 2011), and it cannot be calculated or predicted which of these futures will actually materialize (Beckert, 2013). He suggests that fictional expectations, i.e., “image actors form as they consider future states of the world, the way they visualize causal relations, and the ways they perceive their actions influencing outcomes” (Beckert, 2016, p. 9), reduce uncertainty and enable rational actions. Previous definitions of entrepreneurial visions (e.g., Filion, 2004; Gartner et al., 1992) thus resonate well with this concept of fictional expectations. Beckert pronounces that fictional does not refer to fantasies, but rather “to assessments of future developments that are deemed credible, even though their accuracy cannot be known because they relate to an un-foreknowable future” (Beckert, 2021). Entrepreneurs play an important role in drafting and narrating fictional expectations within the market. Entrepreneurs use fictional expectations as interpretative frames and act “as if” the world and the market would develop as they assume (Gartner et al., 1992). Beckert further supports the idea that the making of the future is not reserved for new ventures, but is a regular response to uncertainty (Beckert, 2021).

In line with a practice theoretical approach, Beckert (2013) argues that the practice of imagining the future for a venture is socially embedded, and hence, entrepreneurial visions cannot be understood in isolation from their context. We can learn from his theory that in their entrepreneurial visions, entrepreneurial actors refer to and assess dominant future discourses, narratives, and stories at the level of the market, field, and society (Beckert, 2016). Fictional expectations thus arise within and respond to a setting of shared norms and expectations towards entrepreneurial behavior (DiMaggio et al. 1991; DiMaggio, 2018). The drafting and narrating of fictional expectations motivate coordinated actions that have real consequences for the venture, market, field, and society (Beckert, 2016). In this perspective, entrepreneurial visions may thus be mental images of future states, but instead of being personal, they are socially constructed.

At the same time, fictional expectations are not determined by their context; rather, actors need to engage in storytelling within what Beckert calls “the politics of expectations” (Beckert, 2013) in order to convince potential customers, employees, investors, suppliers, and other stakeholders of new ideas and future possibilities. While the concept of “externalization” (Wood & McKinley, 2010; Wood et al., 2021) emphasizes a sharing of personal entrepreneurial visions with others, the concept of “politics of expectations” emphasizes that entrepreneurial actors position themselves towards expected or voiced expectations by their stakeholders. In this sense, the dissemination of entrepreneurial visions is political, as actors and stakeholders compete to influence and convince each other, and try to dominate any discourse on imagined futures in such a way that their own expectations prevail (Beckert, 2016). The shared belief that the future will develop in a certain direction and that other (market) actors will behave in a predictable way coordinates the actions and decisions of several actors (Beckert, 2016).

Framing the imagining of the future as a political process enriches an understanding of the discursive negotiation of entrepreneurial visions within entrepreneurial groups. Entrepreneurial visions are not only dynamic, but the content of these visions is essentially interlinked with the arenas in which entrepreneurial actors are positioned towards one another and compete with their fictional expectations.

### The entrepreneurial group as a conditioning interaction arena

Following Preller et al. (2020), and the growing number of contributions on collective engagement in entrepreneurship (Klotz et al., 2014), we argue that entrepreneurial groups play a vital role in the imagining of a future for the venture: the entrepreneurial group serves as an interaction arena, in which group members share and negotiate their observations, assessments, and future ideas—and in this practice constitutes itself as a collective actor. On these grounds, we can reasonably assume that the entrepreneurial group and its specific features (such as size, role distribution) conditions the politics of expectation with regard to a venture’s future. The specifics of small groups, however, remain undertheorized in the entrepreneurship discourse. We thus enrich our theoretical framework with insights from Fine’s (2012) practice-based theory of group action and culture.

Before we do so, however, some clarification around the term entrepreneurial group is needed. The growing research around entrepreneurial teams, new venture teams, founding teams, and entrepreneurial groups has demystified the lone entrepreneur and instead suggested that in the practice of entrepreneuring a small number of people provide financial, labor, or emotional support to drive the venture (Ben-Hafaïedh, 2017; Kamm et al., 1990; Klotz et al., 2014). While this stream of research has yielded important insights on the dynamics of entrepreneuring, conceptual clarity about the social order arising from this activity is lacking (Klotz et al., 2014; Stamm, 2021). Researchers largely agree that the emerging business, as an organization, is distinct from the entrepreneurial team or group, yet there are only a few conceptualizations of this social unit (see, for example, Cooney, 2005; Harper, 2008; Ruef, 2010).

Although the term “entrepreneurial team” is much more common in the discourse, and is often used interchangeably with entrepreneurial groups, we call attention to the conceptual differences of a “team” and a “small group” and view the latter as bringing an essential conceptual advantage for our study: The concept of “team” mostly describes the social relationship between individuals and team boundaries by borrowing from their organizational position (Fine, 2012; Kühl, 2021). Top management teams, founding teams, or new venture teams all presuppose the team to act within an organizational frame and to share organizational outcomes. Definitions of entrepreneurial teams call for team members to be owners or to be involved in the strategic decision making in a (newly emerging) business and thus hold a formal role within an organization (Klotz et al., 2014; Schjoedt & Kraus, 2009). Commitment to an entrepreneurial venture and participation in the practice of drafting an imagined future, however, is not limited to those holding a formal role within an organization. A focus on entrepreneurial teams as a unit of analysis may thus exclude important voices in the practice of imagining the future.

In contrast, a practice-based concept of “small groups” describes social relationships between individuals, based on their joint involvement in a practice (Fine, 2012). Small groups “recognize that they constitute a meaningful social unit, interact on that basis, and are committed to that social unit” (Fine, 2012, p. 21). Hence, entrepreneurial teams and entrepreneurial groups can be identical, when all members involved in the practice of imagining the future hold formal roles, but are most likely not identical, given that additional members emotionally or socially invested in the venture may weigh in during the group level politics of expectations—entrepreneurial groups is thus a broader term than entrepreneurial teams (Schjoedt & Kraus, 2009).

We can learn from Fine’s theory of group action and culture (2012) that entrepreneurial groups construct a shared history and future through continuous reference to their past and prospective venturing activities; they nurture an idioculture, i.e., a system of knowledge, beliefs, and behaviors; and they use their future expectations as orientation for further venturing activities. As interaction arenas, entrepreneurial groups provide spaces for the collective development, appropriation, and interpretation of meanings and objects (Fine, 2012, p. 26). Similar to Beckert, Fine argues that these sets of meanings that group members refer to in their entrepreneurial visions do not need to be constructed anew, but are rather borrowed and pooled from pre-existing sets of meanings and narratives of previous entrepreneurial experiences, other group affiliations, or national culture. Nonetheless, these sets of meanings are group-specific, as the group continuously negotiates meaning, roles (i.e., institutionalized behavior expectations), and the belief in a shared future.

The small group functions as an interaction arena, in which continuous politics of expectations occur and are conditioned by the group. In these processes, the group’s idioculture takes shape and power relations unfold. The (formal) relationships can feature continuous, direct, usually permanent interactions between principally equal partners (symmetry), or the one-sided right to set instructions and assumptions of responsibility (asymmetry) (Stegbauer, 2011, p. 95; Fine, 2012, 21; 25). This structural hierarchy of group members’ role in the organization informs but must not map onto the group’s informality and behavior within the group. The group’s culture and hierarchy (including behavior expectations) can always be challenged, interpreted, or reinvented (Fine, 2012).

For these reasons, we argue that the shared past, i.e., the group’s culture and structural hierarchy, shape how entrepreneurial groups reinforce, draft, and narrate entrepreneurial visions. The entrepreneurial group is not only an important social context for the practice of imagining the future, but is itself a collective actor—the “carrier” of the practice of imagining the future (Gartner et al., 2016, p. 814)—that filters and adopts expectations from its context. Thus, the practice of imagining the future links the agency of the group with its own structures and conditions. Building on this theoretical framework, we strive to conceptualize the ways in which group conditions influence the content of entrepreneurial visions.

## Method

### Sample

In order to study the practice of imagining the future, we turn to German crafts. This important sector of the German economy is influenced by craft conventions and institutions (Diaz-Bone, 2018). Over the decade before the coronavirus pandemic, German crafts experienced positive economic development, although the sector also faced a number of megatrends such as demographic change, changing consumption patterns, European integration, and technological developments (Dürig, 2012). Facing these trends, ventures act under a high degree of ambiguity and uncertainty. Hence, we sample entrepreneurial groups that must position themselves towards these trends and draft entrepreneurial visions for their ventures under the pressure of normative, technological, and market changes, as well as established group routines and structures.

Overall, 12 craft entrepreneurial groups served as our research sites (Table A1). Most of them are entrepreneurial families. As a starting point for our sample, we selected three important trades with one representative profession each: for construction, the plumbing, and heating installation profession; for food, the bakery profession; and for health, dental crafts. Within each profession, we recruited groups attached to businesses that had been in operation for at least 5 years. Following a theoretical sampling approach, we selected new cases on the basis of inductively derived interesting characteristics that appeared relevant, ceteris paribus, in influencing the drafting of entrepreneurial visions in groups (Glaser & Strauss, 2012). We recruited cases that varied in their group composition in terms of group size (dyads, triads, larger), business size (small, medium, large), region (east/west), and succession status (succession y/n).

**Table 1:** Role confidence and dominant set of fictional expectations

Case	Dominant Set of Fictional Expectations	Dimensions of Dominance		
		Volume	Priority	Urgency
High role confidence				
Bread	f.e. for the business	High	High	High
Pretzel	f.e. for the business	Medium	High	Medium
Fruit bread	f.e. for the business	High	High	High
Ginger bread*	f.e. for the business	Medium		High
Tooth	f.e. for the business	High	High	Low
Pontic	f.e. for the business	High	High	High
Dental*	f.e. for the business	Medium	High	Medium
Pipe cutter	f.e. for the business	High	High	Low
Low role confidence				
Pancake	f.e. for the group	High	High	High
Crown*	f.e. for the group	Medium	High	High
Plumbing	f.e. for the group	High	High	High
Oil*	f.e. for the group	Low	High	High

### Data collection

Imagining the future in entrepreneurial groups can occur at any time and place, as our study participants confirmed, e.g., during private breakfasts, dog walks, and holidays. Hence, this practice is unavailable for instantaneous observations. We thus decided for a case study approach centered on group interviews (Bohnsack, 2010; Wimbauer & Motakef, 2017), where groups were stimulated to openly talk about their future expectations.

During group interviews, group members narrate with minimal interviewer instructions. The interviews create a platform where group members can draft their entrepreneurial visions while drawing from their underlying relevancy structures, knowledge horizons, and group dynamics. This helped us gain insight into verbal content and discursive organizations that displayed ongoing informal power dynamics and routines of negotiation (Bohnsack, 2010; Przyborski, 2004). In the first part of the group interview, we asked participants to talk about the history of their venture; in the second part, we stimulated conversations about the future as if it had already happened, using Weick’s “future perfect thinking” exercise (Gioia et al., 2002; Pitsis et al., 2003; Weick, 1995). Both stimuli worked well and generated rich narrations of the history and future of the venture.

Overall, this study draws on a rich data set composed of 12 cases conducted by the second author of this article from 2017 to 2019 in three rounds of sampling. For each case, data collection started with an initial phone interview with one group member to learn about the group composition and the venture(s). This was followed by a face-to-face group interview with all/most members. The author conducted a total of 19 h of interviews with 27 participants. The interviews were transcribed verbatim with an indication of narrative overlaps. In addition, the author collected supplementary documents to reconstruct historical data, including registry entries, company websites, and news articles. For reasons of fact-checking or to resolve open questions, she conducted follow-up interviews when needed.

### Data analysis

Our data analysis followed an iterative, multiple-step approach integrating in-depth inductive case research, documentary method, and visualization techniques.

We started out by reconstructing each venture’s history, drawing from initial interviews, the group interview, and supplementary materials. This gave us a better understanding of the venture’s present, and of key conditions of the entrepreneurial groups, including each member’s current formal and informal roles in the venture. We then moved to a case-by-case application of the documentary method, which is particularly suitable to analyze group interviews (Bohnsack et al., 2010; Przyborski, 2004). The method suggests proceeding in three main steps (for a detailed account of this method, see Bohnsack, 2010): First, we selected the group’s response to the future perfect thinking exercise as the most relevant interview passages from the group interview. Second, we conducted a formulating interpretation for each passage by paraphrasing what group members had said. Here, we carved out the topical structure by differentiating topics and subtopics. Third, we analyzed the formal organization of the passage and reconstructed typical patterns of how group members referred to each other.

Building on these in-depth case insights, we moved to comparative case work on the content of entrepreneurial visions, using a grounded theory largely following the recommendations by Gioia (Gioia et al., 2012). As a base for our coding, we focused on the future passages and results from documentary method analysis (topical structures, narrative patterns) but consulted all the available material. In our first order categorization, we grouped topics into larger categories. In order to arrive at our second order categories, we searched for the underlying meaning and for the context and level of activity. The emerging category system unveiled two sets of fictional expectations on the development of the business and the group, which each consist of macrolevel narratives and actions plans. We further coded for two sets of future orientations engraved in these action plans. These content elements make up the specific configuration of the entrepreneurial visions for each case. We used multiple visualization tools available in MaxQDA to analyze the dominance of certain content elements. For example, we produced color-coded plots for each group’s response to future perfect thinking (Fig. A1), which enabled us to analyze the dominance of business or group fictional expectations in various dimensions (volume, priority, urgency). 

In an iterative process, we related the uncovered content elements and their dominance to group conditions (structure, culture, and discourse organization). We compared color plots, drew diagrams, or used tables to sort the logic between emerging relationships (Strauss & Corbin, 1998). During this process, we continually returned to the specific conditions of entrepreneurial groups. We coded for what we called the role confidence of groups, and through visualizing the discourse organization (see Table A2 for an example), we were able to extract discourse positions for each group member and derive narrative hierarchies for each case. Ultimately, we detected a strong correspondence between, on the one hand, role confidence and the sets of fictional expectations, and, on the other, between the congruence of structural and narrative hierarchies and future orientation. We checked the robustness of these relationships by systematically comparing them with other potential explanatory factors such as industry, group size, group heterogeneity, or succession status. Our multiple case design allowed us to compare and recognize relationships and underlying logical arguments within and across cases (Strike & Rerup, 2016). This procedure provided varied empirical evidence and resulted in a more parsimonious, robust, and generalizable theoretical argument, as well as the formulation of two propositions.

**Table 2:** Congruence of hierarchies and future orientation

Case	Structural hierarchy	Narrative hierarchy	Congruence of hierarchies	Future orientation
Bread	Symmetric	Symmetric	Congruent	Continuing
Pretzel	Asymmetric	Symmetric	Incongruent	Divergent
Fruit	Asymmetric	a/s	Incongruent	Divergent
Bread				
Ginger	Asymmetric	a/s	Incongruent	Divergent
Bread				
Tooth	Symmetric	Symmetric	Congruent	Continuing
Pontic	Asymmetric	Symmetric	Incongruent	Continuing
Dental	Asymmetric	a/s	Incongruent	Divergent
Pipe	Symmetric	Symmetric	Congruent	Continuing
Cutter				

## Findings

### Content elements of entrepreneurial visions

In this section, we present our findings on the content elements of entrepreneurial visions, before relating these contents to group conditions in the following sections. The content of entrepreneurial visions that craft entrepreneurs narrated follows a typical structure and can be categorized in two sets of fictional expectations with two forms of future orientations, which in their combination make up the specific configuration of the entrepreneurial visions for each case.

Entrepreneurial visions for craft ventures are composed of two sets of *fictional expectations* that pertain either to the future development of the business or the entrepreneurial group. We find that each of these sets is composed of references to public discourses on megatrends in the craft sector: respectively, cultural norms of work and working together, which we refer to as macrolevel narratives. These macro-level narratives are used to derive and legitimize action plans to achieve an envisioned future state, enabling the “acting as if” on a business or group level (see Fig. A2).

#### Fictional expectations for business development

This content set refers to all narrations about the future that refer to exploiting and discovering business opportunities (see Table A3a). Typically, macrolevel narratives are connected to imagined ways for maintaining or growing the business. For example, when the group Crown talks about the future role of one of their biggest competitors, they discuss: “[h:] They are going to die. [w:] well, we don’t know that for sure. [h:] Just look at the numbers! … Five years ago, one dentist had 2000 patients, now it’s only 800 patients. His waiting room is almost empty! It’s only a matter of time …. And that is going to be a big advantage for us!” Here, the group refers to the market decline of dental practices as a macrolevel narrative. Other macro-level narratives include trade, economic, or societal change. These narratives are suggested by experts, talked about in business networks, or extrapolated from client behavior and business conventions. Such narratives are often underpinned with statistics and calculative prognoses, as in the above example. They are used to derive actions that need to be taken for the business, such as waiting in the above case, or hiring employees or updating facilities in other cases, or identifying constraints to their entrepreneurial actions (see Table A3a).

#### Fictional expectations for group development

The second content set pertains to all narrations about the way the group will work together in the future (see Table A3b). Typically, institutionalized narratives about life courses and work norms are connected to imagined group compositions and interactions. These expectations for group development are regularly based on the group’s history, culture, and environment. For example, the group Fruit Bread plans to provide the newly entered group member and sons of the bakery’s founder with the opportunity to “develop personal productivity and to […]redesign and implement own ideas.” [xy] The group counters the common narrative of “the old man not giving up the reins” [xy], based on their family’s experience of three generations of successful transfer. Instead, they engage in a narrative of early empowerment when drafting the fictional expectation for the group’s development. Other fictional expectations for group development refer to macro-level narratives about work-life balance or the idea of a second act for retired individuals, and they derive action plans in terms of reducing workloads or planning a group member’s exit (see Table A3b).

Within the set of fictional expectations for business development, there are, however, differences in how the future is presented in terms of the group’s disposition to adapt to change with their action plans, which we refer to *as future orientation*. We distinguish between a continuing and a divergent future orientation (see also Table A4).

#### Continuing future orientation

These narratives highlight how ventures are susceptible to market, sectoral, or society level changes paired with waiting and observing position, signaling a desire for continuity and stability. For example, when addressing new 3D printing technologies in dental crafts, the group Tooth emphasizes that they will adapt these new technologies the same way as they have done in the past: “We don’t necessarily need more technicians to make things, but we need more technology to expand our sales, which has always worked out well so far.” Or when the group Pipe Cutter talks about potentially increasing their number of orders, they prefer stability over expansion: “We are in a good position at the moment, that we can more or less choose the orders, that we really don’t have to fight for every order, but can do the orders we do conscientiously and calmly.” Future expectations are expressed in a hesitant way and/or often refer to the past and the present in order to emphasize the ability to weather change. They envision remaining on the path traveled and reproducing past behaviors.

#### Divergent future orientations

These narratives feature a reported future awareness, including continuous screening of market, sector, or societal level changes, paired with a strong confidence of being able to creatively deal with upcoming problems. Expectations are expressed in a clear, planned, and convincing way. For example, the group Ginger Bread describes the need to emphasize craftsmanship over mass production: “We still have a long way to go, because my grandfather built a large bakery where the goal was to produce as many rye breads and light rolls as possible and that in the meantime, first of all, that's not what I want to do, and secondly, the competition (laughs) is far too great.” Adapting to change and striving for newness is central to this content element. Especially when talking about the venture, the group refers to themselves as entrepreneurial, including the need to keep going, to never stop learning, and to embrace new ways of doing business.

Although in each case these sets of fictional expectations and future orientations are co-present and strongly intertwined, they are distributed unevenly within and across cases. Questions that arise are: Why do fictional expectations for the business become dominant in some cases, while in others they are suppressed? Why are some fictional expectations discussed with a divergent future orientation, while others remain in a continuing mode? In the following, we propose that group conditions play an important part in explaining the patterned ways in which entrepreneurial visions are drafted and narrated.

### Group condition 1: Role confidence and sets of fictional expectations

When talking about the future, in entrepreneurial groups, the set of fictional expectations towards either the business or the group dominates. We propose that role confidence is a strong and plausible explanatory factor for the dominance of one set over another. We use the term role confidence to describe the level of perceived certainty and clarity about the expectations towards each member’s behavior within the group’s future. If role confidence is high, entrepreneurial groups engage in drafting and narrating expectations for business development. If role confidence is low, entrepreneurial groups are preoccupied with drafting and narrating expectations for the group’s development. This relationship turns role confidence into an important condition for a group to be capable of drafting entrepreneurial visions for their business.

For each group, we summarized the role expectations that members had towards one another (Table A5). In contrast to fictional expectations that pertain to anticipated behavior and action plans in an entrepreneurial vision, role expectations pertain to routine behavior within the established group. We find that for some groups, there is a clear division of tasks and responsibilities, a high agreement of behavioral expectations, and/or an awareness of respective positions in the business. Hence, group members can act confidently upon their own and mutual role expectations. For other groups, however, we observe that role expectations are ambiguous, at least for some group members, which creates a situation of uncertainty in working together. Our case studies suggest that in particular, (sudden) group member turnover or anticipated repositioning of group members open windows of lower role confidence within the group. In addition, groups vary in their capacity to gain (or regain) role competence. For example, in the groups Ginger Bread and Crown, a successor has entered the group within the last years, but their level of role confidence is very different. The group Ginger Bread has high role confidence; the successor, for instance, expresses that “Already from the beginning it was clear that he [the father] won’t be doing this forever […].” In contrast, the group Crown is still unsure about each group member’s role expectations, as the following quote of the incumbent illustrates: “We’ll have to wait and see for the next few years, she (successor) has to prove herself first, whether she enjoys it at all.” This group thus has low role confidence.

The level of role confidence corresponds with the set of fictional expectations that dominates the entrepreneurial vision of the entrepreneurial group, as shown in Table 1. The dominance of one set of fictional expectations over another repeats itself in several ways: the sheer volume of talking about topics in one set in comparison to the other set (Bread, Pontic, Inlay, Plumbing), the starting points that interview participants selected in their answers underline the priority given to a set (Crown, Pipe Cutter), and the urgency to clarify topics within the set of future expectations (Pretzel). The strong dominance of the group’s future in some cases is particularly noteworthy, as all entrepreneurial groups operate under high economic uncertainty; hence, drafting a future for the business could be expected. Nonetheless, in a number of cases, issues of working together were so urgent that they dominated the group’s entrepreneurial vision.

In groups where future expectations towards business development dominate the entrepreneurial vision, the distribution of positions and roles within the group are taken for granted and settled (“for sure,” “definitely,” “We talked about it already, it’s set”). Groups with role confidence present a future of continuous working together in reliable and trustworthy relationships and clear role sets. In terms of anticipated member changes, they have a general idea for a future successor or what their collective exit (e.g., as a couple) should look like. They express a strong social identity as a group (“we”) and a profound belief in their common future. Role confidence, however, does not need to be confused with harmony. Rather, the negative features of their working together and role discontent are accepted (for the moment); tension and conflict within these groups remain implicit or are voiced as continuous (subtle and joking) complaints. In these cases, the continuation of the group itself is not questioned, and there is no urgency to draft a future for the group’s development.

In groups where future expectations towards group development dominate the entrepreneurial vision, role confidence was low, meaning that role behavior expectations of each member are uncertain or at least ambiguous (“Let’s see, maybe, we still need to discuss this”). In these cases, we find a strong inward focus: group members observed the alignment of their life plans, including balancing entrepreneurial work with leisure and family affairs. This is the basis on which group members drafted future expectations for the development of the group. These future expectations appeared to be openly discussed and highly urgent. Some groups with low role confidence even explicitly express a lack of the necessary capacity to focus on business development and investments. For example, the daughter in the group Ginger Bread explains: “and he [father] arrived at a point when he didn’t know if it’s going to continue at all and when he didn’t invest in anything anymore. Well, and that’s what we feel right now.” Open questions and uncertainty of the continuation of the group impede a focus on and investment in the future of the business. In other cases, as future expectations for group development are still in the making, the future expectations for the business may range from waiting (keeping things stable) to grooming (polishing the business to attract successors as new members).

### Group condition 2: Hierarchical congruence and future orientation

When zooming in on the fictional expectations for the business, we noticed that craft entrepreneurs tend towards continuing future orientations. They do observe the change in their field and see the necessity to act upon that change, but their action plans are likely to continue on pathways that have already been taken instead of trying new directions or even challenging existing orders. In a field that draws much of its self-identity from craft conventions and routinized ways of doing things, embracing change seems a demanding task. For example, father in group Plumbing explains that he strives to maintain his customer base rather than constantly recruiting new ones in the future “because 20 or 18 years ago, I had to win and convince these customers […] there is so much work in this, so much emotion and long nights when I woke up and wondered how I will do that”. Or the son and successor in group Oil envision moderate growth for their business, “but in any case and that is important to me I want to keep the family character, because it is nice to come into this house and you can feel how everybody belongs here [..] and I do not want this to be so corporate.” Remarkably, we notice that a continuing future orientation is neither specific to the topic discussed nor to the individual member proposing the topic in the future discussion.

In the following, we will argue that the future orientation depends on what we refer to as hierarchical (in)congruence, that is, to the relationship between the structural and narrative hierarchy. It is a discrepancy or incongruence between structural and narrative hierarchies that signals ongoing power shifts within the group and opens a window to express entrepreneurial vision with a divergent orientation.

In each case, entrepreneurial groups take on formal roles within one (or multiple) businesses as owners or managers; they may be employed in that business, or take on informal roles, resulting in a structural hierarchy. In our analysis of the organization of the future discourse produced by the groups, we focused on the discourse positions of each group member, independent of their formal role (see Table A6). We related these positions and thereby revealed narrative hierarchies, which range on a continuum between asymmetrical and symmetrical relations (see Table A7). For example, the combination of a leader and a follower forms a strongly asymmetrical narrative hierarchy that we called “neglected,” as here one person dominates the discourse and others are overlooked (Crown, Pretzel, Pancake). By contrast, in the combination “unisono,” which is a symmetrical narrative hierarchy, all participants talk as equal partners (Pipe Cutter, Tooth).

In the next step, for each case, we have compared the structural hierarchy and the narrative hierarchy and related the resulting (in)congruence to the group’s future orientation.

As Table 2 suggests, we find that a direct translation of the structural hierarchy into the narrative hierarchy (= congruence) corresponds to a continuing future orientation. This relationship is especially striking in cases with strongly asymmetrical structural hierarchies. For example, in the group Crown, formal power is concentrated on the founder, who fully owns the dental business, holds a management position, and is supported by his employed wife. During the interview, the husband takes the narrative role of a dominant leader, while his wife follows. Hence, the asymmetrical structural hierarchy corresponds to the asymmetrical narrative hierarchy (“neglected”). When discussing the expected state interventions into price regularities of private dental services and its threatening influence for the industry, the husband, as founder, ignores the argument of his wife and highlights: “I prepared the company 35 years ago for that, I said it from the beginning that we won’t offer private services and take higher prices, … for me it was logical, and now we will benefit from that and won’t change that.” Similarly, we find a continuing orientation in groups where a symmetrical structural hierarchy is congruent with a symmetrical narrative hierarchy. Here, groups do not reject the change of the business per se; they rather report future challenges (e.g., complete digitalization of the business) and highlight current obstacles (e.g., shortage of skilled workers) that constrain current change. For example, in the group Bread, the father and son share ownership of the business and hold management positions. When drafting and narrating the imagined future for the venture they speak as “partners.” The father’s idea to build a bakery-themed amusement park on the company’s property, and the son’s objection that recruiting the necessary employees will be difficult due to a shortage of skilled workers, are equally heard. The result is that that the visionary idea is put on hold and outvoted, forming a continuing future orientation.

We find an especially strong divergent orientation in such cases, where the (asymmetrical) structural hierarchy does not correspond with a (asymmetrical) narrative hierarchy. For example, in the groups Fruit Bread and Ginger Bread, the successors hold a low status within the asymmetrical structural hierarchy of the group (no shares or official management functions). Nonetheless, in the future discourse, they narrate from the position of a “newcomer” who sees eye to eye with the leader. Both successors openly, but respectfully, criticize the old ways of doing business and suggest action plans for business change. Their challenging of previous business practices and of the structural hierarchy is not suppressed but, with reference to the history and idioculture of the group, even demanded and expected. For example, the father in the group Fruit Bread proudly says: “When I see how committed he is and what and how much he wants to change here, he needs to have the opportunity to reform it and to create his own added value.”

### Model

**Fig. 1:**
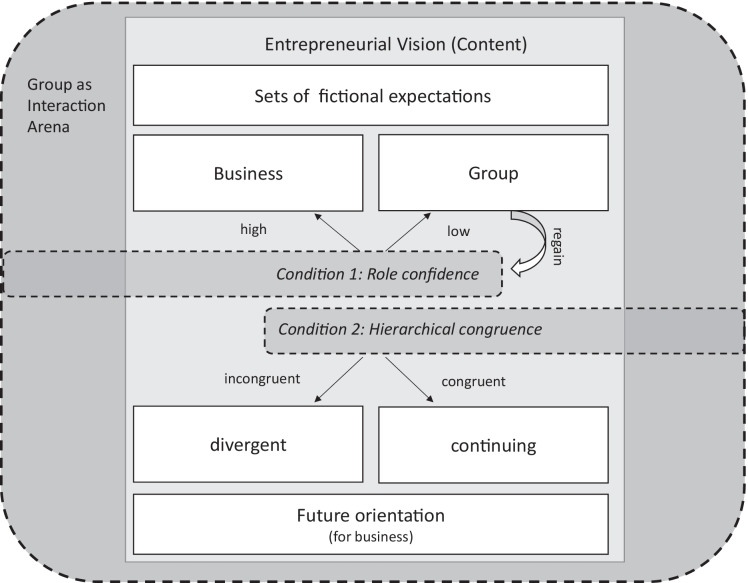
Modeling the relation between the content of entrepreneurial visions and group conditions

Our empirical analysis suggests that the group functions as an important interaction arena in drafting and negotiating entrepreneurial visions. The group filters macro-level narratives on market trends and economic developments, but also on ways of working together, and links these to specific action plans. These entrepreneurial visions for the venture consist of four content elements, as outlined above: two sets of fictional expectations (for the business and the group) and two forms of future orientation. We propose that the specific configuration of these content elements is shaped by the conditions of the group. The following conceptual model depicts the interrelation between group conditions and the content of entrepreneurial visions (Fig. 1).

Group condition 1: Our data suggests that if role confidence is low, i.e., if the group is unclear about the division of tasks or there is ambiguity about group roles, then entrepreneurial groups are likely to draft an entrepreneurial vision with an emphasis on the group’s development (upper right arrow in the model). Consequently, regaining role confidence turns into an important capacity of entrepreneurial groups—otherwise, they will keep going round in circles. If role confidence is high or has been regained, the group is able to draft a future for the business (upper left arrow in the model).

Group condition 2: Among those who are able to draft a future for the business, we observe, across ages and generations, a strikingly high future orientation towards continuity. We identified hierarchical congruence as a second group condition that may be able to explain these differences. We argue that congruence between both forms of hierarchies (lower left arrow in the model) fosters continuing future orientations. If, however, the structural hierarchy does not correspond with the narrative hierarchy, divergent future orientations (lower right arrow in the model) are observed. In the practice of imagining the future, the group condition of incongruent hierarchies seems to allow for and demand the voicing of change narratives.

Establishing the interrelationship between group conditions and the content of entrepreneurial visions serves as a touchstone for a much bolder argument supported by our empirical data: Only groups with role confidence and a narrative hierarchy that challenges structural hierarchy are able to imagine a new future for their business; otherwise, they will remain preoccupied with drafting a future for the group or the same old future for the business.

## Discussion and conclusion

This study begins with the premise that entrepreneurs form an entrepreneurial vision to reduce uncertainty in their venturing activities (Alvarez & Barney, 2007; McMullen & Shepherd, 2006; Wood et al., 2021). The propositions advanced in this paper take an important step towards understanding where this entrepreneurial vision comes from. We point to entrepreneurial groups as interaction arenas for imagining the future, in which the content of entrepreneurial visions is shaped. Overall, we agree with Harrington and Fine (2006) that entrepreneurial groups are an underlying yet important pacemaker of economic dynamics.

Our study enriches current research on the content of entrepreneurial visions by providing a fundamental categorization of content elements. While previous research has aimed at differentiating entrepreneurial visions with regard to organizational development and by their degree of innovativeness (e.g., Kammerlander et al., 2016; Preller et al., 2020; Ruvio et al., 2010), we unveil content elements of entrepreneurial visions that operate on a more fundamental level. Our categorization suggests that aside from drafting fictional expectations for the business, there is a second layer of imagining the future (Andersson 2017; Preller et al., 2020) and a second set of fictional expectations that circulate around the future of a group, including its own distinct cluster of references to macrolevel narratives and action plans. We thereby challenge the assumption that entrepreneurial visions are centered around the development of an organization (Beckert, 2021; Gartner et al., 1994; Garud et al., 2014). As such, our categorization of content elements of entrepreneurial visions, as well as our presented model, widens the perspective of what an entrepreneurial vision is, should, and can entail.

Our presented model interlinks group conditions and the content of entrepreneurial visions. This conceptual link marks an important milestone for an understanding of where entrepreneurial visions come from that moves beyond a personal view of entrepreneurial visions as mental images (Preller et al., 2020; Wood et al., 2021) and acknowledges that entrepreneurial visions are formed in a group political process. We argue, for one, that lacking role confidence in entrepreneurial groups can impede thinking about the future of the business, as the group is preoccupied with its personal relationships and needs to regain confidence in each member’s role (Fine, 2012; Harrington & Fine, 2006; Schäfers, 1999). By drafting future expectations for the group, the group stabilizes a purpose for its “we,” thereby reducing uncertainty on a group level and regaining role confidence. Consequently, regaining role confidence turns into an important capacity of entrepreneurial groups. Role confidence helps groups accept, use, and overcome discrepancies about the business’s future and related uncertainty; it also serves as a linchpin for group members’ motivation to be part of the collective endeavor and assures fundamental trust in the continuation of the collective venture (Harrington & Fine, 2006). Additionally, we argue that narrative hierarchies that challenge structural hierarchies can open a window for embracing actions towards change. As a practice theoretical approach cautions us, a structural hierarchy may be translated into a narrative hierarchy, but the formal power relations must not match the group’s informality (Fine, 2012). In this case, divergent future orientations are observed. In the practice of imagining the future, the group condition of incongruent hierarchies seems to allow for and demand the voicing of change narratives. These change narratives not only challenge business practices but also function as means in the ongoing status and position negotiations within the group (Fine, 2012). In other words, voicing a divergent future orientation can be interpreted as an act of proving oneself and challenging group structures. This link between hierarchical congruence and future orientation reminds us that to be enacted, fictional expectations with a divergent future orientation must be heard and accepted within the group—otherwise, they will remain “ineffective dreams” (Beckert, 2016).

These insights stand on a theoretically enriched understanding of entrepreneurial visions that deliberately connects imagining the future to the discourses on entrepreneurship as practice, entrepreneurial visions, and entrepreneurial groups. The generated findings now reflect back into these different discourses, which are likely to be impacted by our theorizing.

First, we connect to an EaP perspective by establishing imagining the future as an important practice in venturing. From this perspective, we can show how an entrepreneurial vision is socially constructed in this practice (Gartner et al., 2016; Gross et al., 2014; Thompson et al., 2020). We adopt a method that adheres to a practice approach and allows us to study imagining the future in situ (Chalmers & Shaw, 2017). Our findings contribute to an EaP perspective by highlighting the structuring power of entrepreneurial groups as a particular contextual factor in imagining the future (Thompson et al., 2020). Following Clerq and Voronov (2009), we argue that imagining that future is a “socially embedded process connected to entrepreneurs’ positions in structures of power relations” (p. 395) and point to the specific character of groups in which this positioning occurs. In contrast to existing EaP approaches that strongly emphasize the performance of activities during venturing (e.g., Johannisson, 2011; Steyaert, 2007), we call attention to the ostensive dimensions of practice (Feldman & Pentland, 2003) and how the group structure is key to understanding “imagining the future” and its drafted entrepreneurial vision.

Second, we contribute to the discourse on entrepreneurial visions by connecting to the complex sociological theory of imagined futures (Beckert, 2013, 2016). This is in contrast to current approaches to entrepreneurial visions that start at the individual level (Brush, 2008; O’Connell et al. 2011; Miller & Le Breton-Miller, 2017). Importantly for the question of where do entrepreneurial visions come from is that the theory of imagined futures elevates the idea of “externalization” (Wood et al., 2021) to a more complex understanding of “politics of expectations” (Beckert, 2016). We indicate that entrepreneurial visions are constructed in entrepreneurial groups, but not in isolation, and show strong referencing to macrolevel narratives available in market, industry, and societal discourses (Beckert, 2016; Fine, 2012). In drafting and narrating their entrepreneurial vision, entrepreneurs rely on their own experience, but even more importantly on what they hear in the news, in industry associations, and from colleagues, friends, and trusted advisors (Harrington & Strike, 2018; Strike & Rerup, 2016; Welter et al., 2016). We illustrate how ideas about an entrepreneurial vision are vetted within the entrepreneurial group: propositions are formulated, elaborated upon, and critiqued, and conclusions are drawn within the circle of group members. In the practice of imagining the future, power dynamics are enacted (Fine, 2012; Wohlrab-Sahr, 2018). The right to speak, counter, conclude, etc., shapes narrative roles and forms narrative hierarchies. These observations suggest that not all ideas can be heard and have equal weight within groups. Hence, we emphasize the group political aspects of imagining the future as a relevant element in understanding where entrepreneurial visions come from.

Third, our study contributes to current research on entrepreneurial teams and groups (Ben-Hafaïedh, 2017; Klotz et al., 2014; Preller et al., 2020). For one, this study advances and applies a small group approach to entrepreneurial activity. Using small groups rather than teams as unit of analysis echoes Harper’s (2008) call to fully grasp the collective engagement in venturing activities rather than restricting it to those formally involved in the emerging and maintained business. By interviewing small groups rather than teams, the importance of informal group membership in narrative discourse about the future became evident. Without this broader concept, a discovery of hierarchical congruence as group condition shaping the content of an entrepreneurial vision would not have been possible. The integrative approach of entrepreneurial groups indicates a fruitful direction for further conceptual development in entrepreneurship research that could, for example, examine role assignment, power relations, or idioculture. For another, our study draws attention to unfolding group dynamics in situ and thus sheds light on the mediating effects of groups. Klotz et al. (2014) suggest that team processes and emergent team states mediate between the input and output of entrepreneurial teams, and call for more research on the interdependence of such mediating factors. Our study both enrich an understanding of imagining the future as a particular group process and suggests role confidence and hierarchical congruence to be relevant emergent states. Our propositions offer a conceptualization of the interrelation between both. In this sense, our study explores intermediary mechanisms that foster a more precise understanding of how entrepreneurial groups can be effective.

As with all qualitative research, our findings are limited in their generalizability. Future research should test the proposed relationships between group conditions and content elements of entrepreneurial visions in a larger sample, including a range of sectors. The presented findings offer important directions to operationalize the content elements of entrepreneurial visions, as well as role confidence and hierarchical congruence. The development and testing of an adequate scale to measure both group conditions seem promising next steps. Although conducted in an everyday entrepreneurial context, our findings may also inspire research on new venture teams, given that these teams are in the midst of establishing their group’s role confidence and structures (Katz, 1993). As indicated above, the utilized theoretical framework and presented propositions also allow for future research that engages in a theoretical advancement of a practice-based small group approach in entrepreneurship.

Beyond academia, our findings on the link between group conditions and the content of entrepreneurial visions have implications for strategic management in established ventures. In order to engage in a strategic drafting of future plans for their business, entrepreneurial groups need to have role confidence and incongruent hierarchies. Consequently, we suggest that an initial analysis of group conditions—both in strategic management consultancy as well as in the self-reflection of entrepreneurial groups—may be helpful in assessing whether a group is at all receptive to drafting and narrating fictional expectations for their venture. As such, our categorization of the content of imagined futures can serve as a litmus test on the capacity of entrepreneurial groups to engage in drafting an innovative entrepreneurial vision for a business.

## Supplementary Information

Below is the link to the electronic supplementary material.

Supplementary file1 (DOCX 874 KB)

## Data Availability

All interview materials have been transcribed. The anonymity of the participants has been guaranteed; hence, these materials are not publicly available. An anonymized version is available upon request.

## Code availability

N.A.

## References

[CR1] Aldrich, H. E., & Fiol, C. M. (1994). Fools rush in? The institutional context of industry creation. *The Academy of Management Review,**19*(4), 645–670.

[CR2] Alvarez, S. A., & Barney, J. B. (2007). Discovery and creation: Alternative theories of entrepreneurial action. *Strategic Entrepreneurship Journal,**1*, 11–26. 10.1002/sej.4

[CR3] Andersson, J. (2017). The power of the future. Review Symposium. Jens Beckert 2016, Imagined futures, fictional expectations and capitalist dynamics. Harvard University Press. Socio-Economic Review, 15(1), 255–258.

[CR4] Ashford, S. J., Wellman, N., Sully de Luque, M., de Stobbeleir, K. E., & Wollan, M. (2018). Two roads to effectiveness: CEO feedback seeking, vision articulation, and firm performance. *Journal of Organizational Behavior,**39*, 82–95. 10.1002/job.2211

[CR5] Barbera, F., Stamm, I., DeWitt, R-L. (2018) The development of an entrepreneurial legacy. Exploring the role of anticipated futures in transgenerational entrepreneurship. *In: Family Business Review, 31*(3), S. 352–378. 10.1177/0894486518780795

[CR6] Baum, J. R., Locke, E. A., & Kirkpatrick, S. A. (1998). A longitudinal study of the relation of vision and vision communication to venture growth in entrepreneurial firms. *Journal of Applied Psychology,**83*, 43–54. 10.1037/0021-9010.83.1.43

[CR7] Beckert, J. (2013). Imagined futures: Fictional expectations in the economy. *Theory and Society,**42*, 219–240. 10.1007/s11186-013-9191-2

[CR8] Beckert, J. (2016). *Imagined futures: Fictional expectations and capitalist dynamics*. Harvard University Press.

[CR9] Beckert, J. (2017a). Die Historizität fiktionaler Erwartungen. MPIfG Discussion paper 17/18.

[CR10] Beckert, J. (2017b). Woher kommen Erwartungen? Die soziale Strukturierung imaginierter Zukünfte. MPIfG Discussion Paper 17/17.

[CR11] Beckert, J. (2021). The firm as an engine of imagination: Organizational prospection and the making of economic futures. *Organization Theory,**2*, 263178772110057. 10.1177/26317877211005773

[CR12] Ben-Hafaïedh, C. (2017). Entrepreneurial teams research in movement. In C. Ben-Hafaïedh & T. M. Cooney (Eds.), *Research Handbook on Entrepreneurial Teams: Theory and Practice* (pp. 11–44). Edward Elgar Publishing.

[CR13] Bird, B. J. (1988). Implementing entrepreneurial ideas: The case for intention. *Academy of Management Journal,**13*, 442–453. 10.5465/amr.1988.4306970

[CR14] Bohnsack, R. (2010). Documentary method and group discussions. In R. Bohnsack, N. Pfaff, & W. Weller (Eds.), *Qualitative analysis and documentary method in international educational research* (pp. 99–124). Barbara Budrich Verlag.

[CR15] Bohnsack, R., Przyborski, A., & Schäffer, B. (Eds.). (2010). *Das Gruppendiskussionsverfahren in der Forschungspraxis* (2nd ed.). Budrich.

[CR16] Brush, C. G. (2008). Pioneering strategies for entrepreneurial success. *Business Horizons,**51*, 21–27. 10.1016/j.bushor.2007.09.001

[CR17] Chalmers, D. M., & Shaw, E. (2017). The endogenous construction of entrepreneurial contexts: A practice-based perspective. *International Small Business Journal,**35*, 19–39. 10.1177/0266242615589768

[CR18] de Clercq, D., & Voronov, M. (2009). Toward a practice perspective of entrepreneurship. *International Small Business Journal: Researching Entrepreneurship,**27*, 395–419. 10.1177/0266242609334971

[CR19] Cooney, T. (2005). What is an entrepreneurial team. *International Small Business Journal,**23*(3), 226–235.

[CR20] Diaz-Bone, R. (2018). *Die “Economie des conventions”: Grundlagen und Entwicklungen der neuen französischen Wirtschaftssoziologie*. Springer VS.

[CR21] DiMaggio, P., & Powell, W. (Eds.). (1991). The new institutionalism in organizational analysis. Chicago: the University of Chicago press.

[CR22] DiMaggio, P. J. (2018). Our faith-based economy. *Distinktion: Journal of Social Theory*, 19, 328–335. doi:10.1080/1600910X.2018.1452769.

[CR23] Downing, S. (2005). The social construction of entrepreneurship: Narrative and dramatic processes in the coproduction of organizations and identities. *Entrepreneurship Theory & Practice,**29*, 185–204. 10.1111/j.1540-6520.2005.00076.x

[CR24] Dürig, W. (2012). Entwicklung der Märkte des Handwerkes und betriebliche Anpassungserfordernisse. Teil 1: Analyse. Endbericht - November 2012. (RWI Projektberichte).

[CR25] Ensley, M. D., Pearson, A., & Pearce, C. L. (2003). Top management team process, shared leadership, and new venture performance: A theoretical model and research agenda. *Human Resource Management Review,**13*, 329–346. 10.1016/S1053-4822(03)00020-2

[CR26] Feldman, M. S., & Pentland, B. T. (2003). Reconceptualizing organizational routines as source of flexibility and change. Administrative Science Quarterly. 10.2307/3556620.

[CR27] Filion, L. J. (1991). Vision and relations: Elements for an entrepreneurial metamodel. *International Small Business Journal: Researching Entrepreneurship,**9*, 26–40. 10.1177/026624269100900202

[CR28] Filion, L. J. (2004). Operators and visionaries: Differences in the entrepreneurial and managerial systems of two types of entrepreneurs. *International Journal of Entrepreneurship and Small Business,**1*, 35–55. 10.1504/IJESB.2004.005376

[CR29] Fine, A. (2012). *Tiny publics: A theory of group action and culture (Russell Sage Foundation series on trust)*.

[CR30] Gartner, W. B. (2007). Entrepreneurial narrative and science of imagination. *Journal of Business Venturing,**22*, 613–627. 10.1016/j.jbusvent.2006.10.003

[CR31] Gartner, W. B. (Ed.). (2016). *Entrepreneurship as organizing: Selected papers of William B. Gartner*. Edward Elgar Publishing.

[CR32] Gartner, W. B., Bird, B. J., & Starr, J. A. (1992). Acting as if: Differentiating entrepreneurial from organizational behavior. *Entrepreneurship Theory & Practice,**16*(3), 13–31.

[CR33] Gartner, W. B., Shaver, K. G., Gatewood, E., & Katz, J. A. (1994). Finding the entrepreneur in entrepreneurship. *Entrepreneurship Theory & Practice,**18*, 5–10. 10.1177/104225879401800301

[CR34] Gartner, W. B., Stam, E., Thompson, N., & Verduyn, K. (2016). Entrepreneurship as practice: Grounding contemporary practice theory into entrepreneurship studies. *Entrepreneurship & Regional Development,**28*, 813–816. 10.1080/08985626.2016.1251736

[CR35] Garud, R., Schildt, H. A., & Lant, T. K. (2014). Entrepreneurial storytelling, future expectations, and the paradox of legitimacy. *Organization Science,**25*, 1479–1492. 10.1287/orsc.2014.0915

[CR36] Gioia, D. A., Corley, K. G., & Fabbri, T. (2002). Revising the past (while thinking in the future perfect tense). *Journal of Organizational Change Management,**15*, 622–634. 10.1108/09534810210449532

[CR37] Gioia, D. A., Corley, K. G., & Hamilton, A. L. (2012). Seeking qualitative rigor in inductive research: Notes on the Gioia Methodology. *Organizational Research Methods,**16*, 15–31. 10.1177/1094428112452151

[CR38] Glaser, B. G., & Strauss, A. L. (2012). *The discovery of grounded theory: Strategies for qualitative research* (7th ed.). Aldine Pub. Co.

[CR39] Gross, N., Carson, D., & Jones, R. (2014). Beyond rhetoric: Re-thinking entrepreneurial marketing from a practice perspective. *Journal of Research in Marketing and Entrepreneurship,**16*, 105–127. 10.1108/JRME-01-2014-0003

[CR40] Harper, D. A. (2008). Towards a theory of entrepreneurial teams. *Journal of Business Venturing,**23*(6), 613–626.

[CR41] Harrington, B., & Fine, G. A. (2006). Where the action is: Small groups and recent developments in sociological theory. *Small Group Research,**37*(1), 4–19.

[CR42] Harrington, B., & Strike, V. M. (2018). Between kinship and commerce: Fiduciaries and the institutional logics of family firms. *Family Business Review,**31*, 417–440. 10.1177/0894486518780868

[CR43] Haynie, J. M., Shepherd, D., Mosakowski, E., & Earley, P. C. (2010). A situated metacognitive model of the entrepreneurial mindset. *Journal of Business Venturing,**25*, 217–229. 10.1016/J.JBUSVENT.2008.10.001

[CR44] Jaskiewicz, P., Combs, J., & Rau, S. (2015). Entrepreneurial legacy: Toward a theory of how some family firms nurture transgenerational entrepreneurship. *Journal of Business Venturing,**30*, 29–49. 10.1016/j.jbusvent.2014.07.001

[CR45] Johannisson, B. (2011). Towards a practice theory of entrepreneuring. *Small Business Economy,**36*, 135–150. 10.1007/s11187-009-9212-8

[CR46] Johansson, A. (2004). Narrating the entrepreneur. *International Small Business Journal,**22*, 273–293. 10.1177/0266242604042379

[CR47] Kamm, J., Shuman, J. C., Seeger, J. A., & Nurick, A. J. (1990). Entrepreneurial teams in new venture creation: A research agenda. *Entrepreneurship Theory & Practice,**14*(4), 7–17.

[CR48] Kammerlander, N., Dessi, C., Bird, M., Floris, M., & Murru, A. (2016). The impact of shared stories on family firm innovation: A multi-case study. *Family Business Review,**28*, 332–354. 10.1177/0894486515607777

[CR49] Kantabutra, S. (2009). Toward a behavioral theory of vision in organizational settings. *Leadership & Organization Development Journal,**30*, 319–337. 10.1108/01437730910961667

[CR50] Katz, J. A. (1993). The dynamics of organizational emergence: A contemporary group formation perspective. *Entrepreneurship Theory & Practice,**17*, 97–101. 10.1177/104225879301700210

[CR51] Keating, A., & McLoughlin, D. (2010). The entrepreneurial imagination and the impact of context on the development of a new venture. *Industrial Marketing Management,**39*, 996–1009. 10.1016/j.indmarman.2010.06.019

[CR52] Kier, A. S., & McMullen, J. S. (2018). Entrepreneurial imaginativeness in new venture ideation. *Academy of Management Journal,**61*, 2265–2295. 10.5465/amj.2017.0395

[CR53] Klotz, A. C., Hmieleski, K. M., Bradley, B. H., & Busenitz, L. B. (2014). New venture teams: A review of the literature and roadmap for future research. *Journal of Management,**40*, 226–255. 10.1177/0149206313493325

[CR54] Knockaert, M., Ucbasaran, D., Wright, M., & Clarysse, B. (2011). The relationship between knowledge transfer, top management team composition, and performance: The case of science-based entrepreneurial firms. *Entrepreneurship Theory and Practice,**35*, 777–803. 10.1111/j.1540-6520.2010.00405.x

[CR55] Kühl, S. (2021). Die folgenreiche Verwechslung von Teams, Cliquen und Gruppen. Gruppe. Interaktion. Organisation. Zeitschrift für Angewandte Organisationspsychologie (GIO). 10.1007/s11612-021-00576-8.

[CR56] Lounsbury, M., & Glynn, M. A. (2001). Cultural entrepreneurship: Stories, legitimacy, and the acquisition of resources. *Strategic Management Journal,**22*, 545–564. 10.1002/smj.188

[CR57] McKelvie, A., Haynie, J. M., & Gustavsson, V. (2011). Unpacking the uncertainty construct: Implications for entrepreneurial action. *Journal of Business Venturing,**26*, 273–292. 10.1016/j.jbusvent.2009.10.004

[CR58] McMullen, J. S., & Shepherd, D. A. (2006). Entrepreneurial action and the role of uncertainty in the theory of the entrepreneur. *Academy of Management Review,**31*, 132–152. 10.5465/amr.2006.19379628

[CR59] Miller, D., & Le Breton-Miller, I. (2017). Sources of entrepreneurial courage and imagination: Three perspectives, three contexts. *Entrepreneurship Theory and Practice,**41*, 667–675. 10.1111/etap.12281

[CR60] O’Connell, D., Hickerson, K., & Pillutla, A. (2011). Organizational visioning: An integrative review. *Group & Organization Management,**36*, 103–125. 10.1177/1059601110390999

[CR61] Packard, M. D., Clark, B. B., & Klein, P. G. (2017). Uncertainty types and transitions in the entrepreneurial process. *Organization Science,**28*, 840–856. 10.1287/orsc.2017.1143

[CR62] Pitsis, T., Clegg, S., Marosszeky, M., & Rura-Polley, T. (2003). Constructing the olympic dream: A future perfect strategy of project management. *Organanization Science,**14*(5), 574–590.

[CR63] Preller, R., Patzelt, H., & Breugst, N. (2020). Entrepreneurial visions in founding teams: Conceptualization, emergence, and effects on opportunity development. *Journal of Business Venturing,**35*, 105914. 10.1016/j.jbusvent.2018.11.004

[CR64] Przyborski, A. (2004). Gesprächsanalyse und dokumentarische Methode: Qualitative Auswertung von Gesprächen, Gruppendiskussionen und anderen Diskursen. Wiesbaden: VS Verlag für Sozialwissenschaften.

[CR65] Ruef, M. (2010). *The entrepreneurial group: Social identities, relations, and collective action (Kauffman Foundation series on innovation and entrepreneurship)*. Princeton University Press.

[CR66] Ruvio, A., Rosenblatt, Z., & Hertz-Lazarowitz, R. (2010). Entrepreneurial leadership vision in nonprofit vs. for-profit organizations. *The Leadership Quarterly,**21*, 144–158. 10.1016/J.LEAQUA.2009.10.011

[CR67] Schäfers, B. (1999). Entwicklung und Grundlegung der Gruppensoziologie. In B. Schäfers (Ed.), Einführung in die Gruppensoziologie: Geschichte, Theorien, Analysen (3rd ed., pp. 19–36, Vol. 996). Wiesbaden: Quelle und Meyer.

[CR68] Schjoedt, L., & Kraus, S. (2009). Entrepreneurial teams: Definition and performance factors. *Management Research News,**32*(6), 513–524.

[CR69] Stamm, I. (2021) Groups matter: Social embeddedness of entrepreneurial activity. In: A. Maurer und Andrea Maurer (Hg.):* Handbook of Economic Sociology for the 21st Century. New Theoretical Approaches, Empirical Studies and Developments*. Cham: Springer VS; Springer International Publishing, S. 253–268.

[CR70] Stegbauer, C. (2011). Reziprozität: Einführung in die soziale Formen der Gegenseitigkeit (2nd ed.). VS Verlag.

[CR71] Steyaert, C. (2007). ‘Entrepreneuring’ as a conceptual attractor?: A review of process theories in 20 years of entrepreneurship studies. *Entrepreneurship & Regional Development,**19*, 453–477. 10.1080/08985620701671759

[CR72] Strauss, A. L., & Corbin, J. (1998). *Basics of qualitative research*. Sage Publications.

[CR73] Strike, V. M., & Rerup, C. (2016). Mediated sensemaking. *Academy of Management Journal,**59*, 880–905. 10.5465/amj.2012.0665

[CR74] Thompson, N. A., Verduijn, K., & Gartner, W. B. (2020). Entrepreneurship-as-practice: Grounding contemporary theories of practice into entrepreneurship studies. *Entrepreneurship & Regional Development,**32*, 247–256. 10.1080/08985626.2019.1641978

[CR75] Uprichard, E. (2011). Narratives of the future: complexity, time, and temporality. In M. Williams & W. P. Vogt (Eds.), The SAGE Handbook of Innovation in Social Research Methods (pp. 103–119). Los Angeles: SAGE.

[CR76] Weick, K. E. (1995). Sensemaking in organizations (Foundations for organizational science). Thousand Oaks, Calif: SAGE.

[CR77] Welter, F., Baker, T., Audretsch, D. B., & Gartner, W. B. (2016). Everyday entrepreneurship: A call for entrepreneurship research to embrace entrepreneurial diversity. *Entrepreneurship Theory and Practice,**41*, 311–321. 10.1111/etap.12258

[CR78] Wimbauer, C., & Motakef, M. (2017). Das Paarinterview in der soziologischen Paarforschung: Method(olog)ische und forschungspraktische Überlegungen. Forum: Qualitative Sozialforschung. 10.17169/fqs-18.2.2671.

[CR79] Wohlrab-Sahr, M. (2018). Das Kollektive oder das Sozial-Interaktive? Was bekommt man beim Paar- und Familieninterviews zu sehen? Ad-hoc Gruppe Lebenszusammenhänge und Ungleichheit erforschen. Methode und Praxis von Paar-, Familien- und Haushaltsinterviews. Universität Göttingen, Göttingen, September, 26.

[CR80] Wood, M. S., Bakker, R. M., & Fisher, G. (2021). Back to the future: A time-calibrated theory of entrepreneurial action. *Academy of Management Review,**46*, 147–171. 10.5465/amr.2018.0060

[CR81] Wood, M. S., & McKinley, W. (2010). The production of entrepreneurial opportunity: A constructivist perspective. *Strategic Entrepreneurship Journal,**4*, 66–84. 10.1002/sej.83

