# Supplementary material for: Group conditions for entrepreneurial visions: role confidence, hierarchical congruences, and the imagining of future in entrepreneurial groups
Source: Small Bus Econ (Dordr). 2021 Nov 30;59(3):1023–41. doi: 10.1007/s11187-021-00566-6 (PMC8631563; doi:10.1007/s11187-021-00566-6)
Supplement: Supplementary file 1 — Supplementary file1 (DOCX 874 KB) [file 11187_2021_566_MOESM1_ESM.docx]

**Online Supplementary Material**

Table A1: Overview of Cases

| Case | Group Characteristics Overview | | | | | | | Business Characteristics Overview | | | |
| --- | --- | --- | --- | --- | --- | --- | --- | --- | --- | --- | --- |
|  | Name | Group Members | Age of Members | Group Ties | Formal roles in b | Structural Hierarchy |  | Industry | Size of  Business | Region | Year  Founded |
| C1 | Bread | **1***  **2***  3 | 56  34  76 | Family | Owner-manager (51%)  Owner-manager (49%)  Manager (CEO) | Symmetric |  | Bakery | Large | Saxony | 1913 |
| C2 | Pretzel | **1***  **2***  3  **4*** | 39  60  65  47 | Family | Owner-manager (68%),  Owner (16%)  Owner (16%)  Manager (CEO) | Asymmetric |  | Bakery | Large | Bavaria | 1895 |
| C3 | Pancake | **1***  2  3  **4***  5  6  7 | 54  52  28  26  28  78  78 | Family | Owner-manager (100%)  Employee (wife),  Helping family member (d.)  Employee (son)  Helping family member  Helping family member  Helping family member | Asymmetric |  | Bakery | Small | Saxony-Anhalt | 1933 |
| C4 | Fruit  Bread | **1***  2  **3***  **4***  5 | 54  52  25  81  75 | Family | Owner-manager (100%);  Employee  Employee  Helping family member  Helping family member | Asymmetric |  | Bakery | Medium  (120 employees) | Bavaria | 1996 |
| C5 | Ginger  Bread | 1  2 | 59  24 | Family | Owner-manager (100%)  Manager | Asymmetric |  | Bakery | Medium | Bavaria | 1902 |
| C6 | Tooth | 1  2 | 58  34 | Friends | Owner-manager (51%)  Owner-manager (49%) | Symmetric |  | Dental | Small | Berlin | 2012 |
| C7 | Crown | 1  2 | 64  49 | Couple | Owner-manager (100%)  Employee | Asymmetric |  | Dental | Large | Berlin | 1993 |
| C8 | Pontic | 1  2 | 51  51 | Couple | Owner-manager (100%)  Manager (CEO) | Asymmetric |  | Dental | Small | Saxony-Anhalt | 1987 |
| C9 | Dental | **1***  2  **3***  4  5 | 58  60  27  25  30 | Family | Owner manager (100%)  Owner manager (100%)  Owner manager (100%)  Employee  Helping familiy member | Asymmetric |  | Dental | 3 small b. | Berlin | 1990, 2005, 2015 |
| C10 | Pipe Cutter | 1  2 | 37  33 | Siblings | Owner-manager (50%)  Owner-manager (50%) | Symmetric |  | Plumbing and Heating | Small | Berlin | 2012 |
| C11 | Plumbing | **1***  **2***  **3*** | 50,  46  21 | Family | Owner- manager (100%)  Employee  Deputy manager | Asymmetric |  | Plumbing and Heating | Small | Berlin | 1997 |
| C12 | Oil | **1***  2  **3***  4  5  6 | 57,  77  28  ?  ?  50 | Family  Friends | Owner-manager (50%) Owner (50%)  Deputy manager  Manager (CEO)  Manager  Employee | Asymmetric |  | Plumbing and Heating | Large | Berlin | 1962 |

******these members of the group participated in the interviews*

Table A2: Exemplary analysis of the discourse organization in Case Oil representing a consecutive narrative hierarchy

| **Participant A (narrative role: leader)** | **Reference** | **Participant B (narrative role: newcomer)** |
| --- | --- | --- |
| **P1_A: I have a plan** |  |  |
| E1_P1: successful organization structure; 1st management can go on vacation, everything is going well, son set as future managing director |  |  |
| **P2_A: Lack of skilled workers - belief that the issue will soon be resolved** |  |  |
| E1_P2: past, great commitment to education, he has laid important foundations |  |  |
| K1_P2: „well, yeah“ |  |  |
| **P3_A: My plan has worked (10 years): I can proudly look back on the continuation of the company (eagle figure)** |  |  |
| E1_P3: Proud of continuation by son, expectations of him to prepare the next generation for entrepreneurship |  |  |
| E2_P3: Proud that son has become an entrepreneur, rejection of idea of work-life balance |  |  |
| E3_P3: Possibilities but also limits of digitization in the crafts enterprise |  |  |
| K1_P3: Company continues to run successfully (stable, smoothly) even without me with reliability and sense of duty - that's enough for me and makes me happy (85 - 93) |  |  |
| **P4_A: I'll be gone soon, have done a final tour de force for the company (location)** |  |  |
|  |  | E2(R)_P5: Laughter |
| E1_P4: communicated his plan clearly, created preparation and conditions for future growth, ideal conditions for employees (in terms of the house) |  |  |
| K_P4: Yeah |  |  |
|  |  | **P5_B: Yes I agree with you (120)** |
|  |  | E1_P5: in 10 years the company will continue to run successfully and solidly = my highest goal (120-122) |
|  |  | K_P5: laugh „in any case“ (122) |
|  |  | **P6_B: Shortage of skilled workers remains an important topic for business in the coming years (126 - 129)** |
|  |  | E1_P6: we are currently doing everything we can to make sure that this will not be a problem (129) |
|  |  | K_P6: but: Staff shortage seen as cause for lack of growth (also in other companies of the same size) (130 - 134) |
|  |  | **P7_B: yes to growth, but limited (134 - 135)** |
|  |  | E1_P7: Family character can only be maintained as long as a pleasant working atmosphere is maintained ("finding each other") (135 - 138) |
|  |  | E2_P7: because fun at work is already important to me, rejection of corporate structure (138 - 140) |
|  |  | E3_P7: Employees where generations are already involved (145 - 147) |
| E4_P7: Consent (149) |  |  |
|  |  | E5_P7: Three generations (151) |
|  |  | K_P7: Desire and pride in the long term and continuation (instead of growth) |
|  |  | **P8_B: We must continue to develop ourselves (152 - 153)** |
|  |  | E1_P8: digital topics  + Process optimization for employees on site. (153 – 164) |
|  |  | K_P8: uhm, yeah (168) |
|  |  | **P9_B: Home location exciting city (168 - 170)** |
|  |  | E1_P9: Restricted by political conditions and situation, can become dangerous (170 - 192) |
|  |  | K_P9: Duty to deal with these political conditions |
|  |  | **P10_B: Future technologies as an opportunity (194 - 196)** |
|  |  | E1_P10: Market of the future, positive development (196 - 203) |
|  |  | E2_P10: We want to remain at the cutting edge of technology  - his drive (203 - 215) |
|  |  | K_P10: yeah (215) |
|  |  | **P11_B: Uncertainty about strategic goals** |
|  |  | E1_P11: Looking left and right for possible further developments of the business field, but he has no concrete examples |
|  |  | E2_P11: We have always tried and tried, failing and fighting is part of it (223 - 230) |
|  |  | K_P11: „yeah“ (234) |
| **P12_A: Family character is important to employees: I underestimated that (238 - 241)** |  |  |
| E1_P12: Differentiation to industrial companies (245 - 247) |  |  |
| E2_P12: Leadership mentality: boss should be tangible |  |  |
|  |  | E3_P12: Father lives it, example for problems (259 - 264) |
|  |  | E4_P12: Differentiation: instead of one-sidedness, rather dialogue and closeness to employees, difference between past (father) and present (him) |
|  |  | K_P12: this works well -> ritual conclusion, since only one "mhm" comes from the father |

**Explanation of the arrows:**

The arrows visualize the ways of referring to positions of other group members:

black: neutral or differentiating reference

green: approving reference (appreciative)

orange: negative reference (not appreciative)

Continiuous line: direct reference (recording)

dotted line: indirect reference

**Numberting of topics**

Type of topic (letter) Sequence number (number) _ Reference to named topics

P: Proposition V: Validation

TP: Transposition R: Ratification

E: Elaboration D: Differentiation

K: Conclusion

Figure A1: Visualization plot of the interview passage after the future perfect thinking stimulus generated via MaxQDA


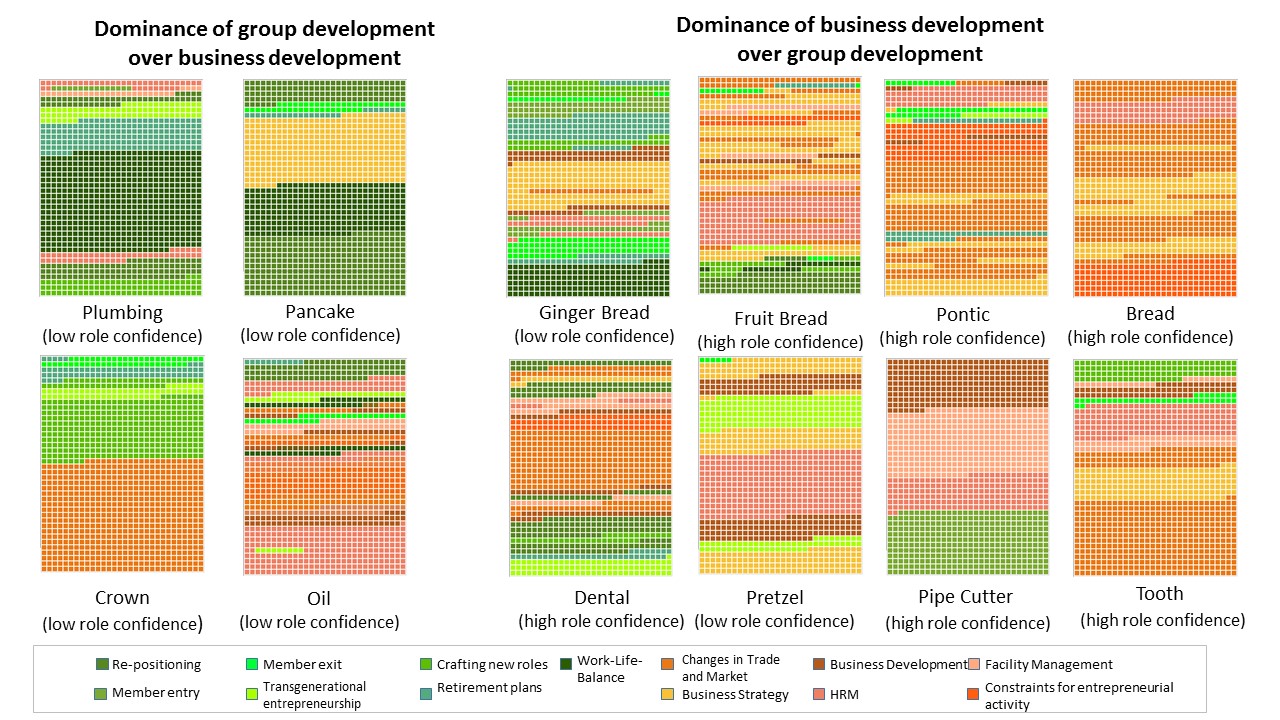


Figure A2: Two types of fictional expectations as content elements of entrepreneurial visions


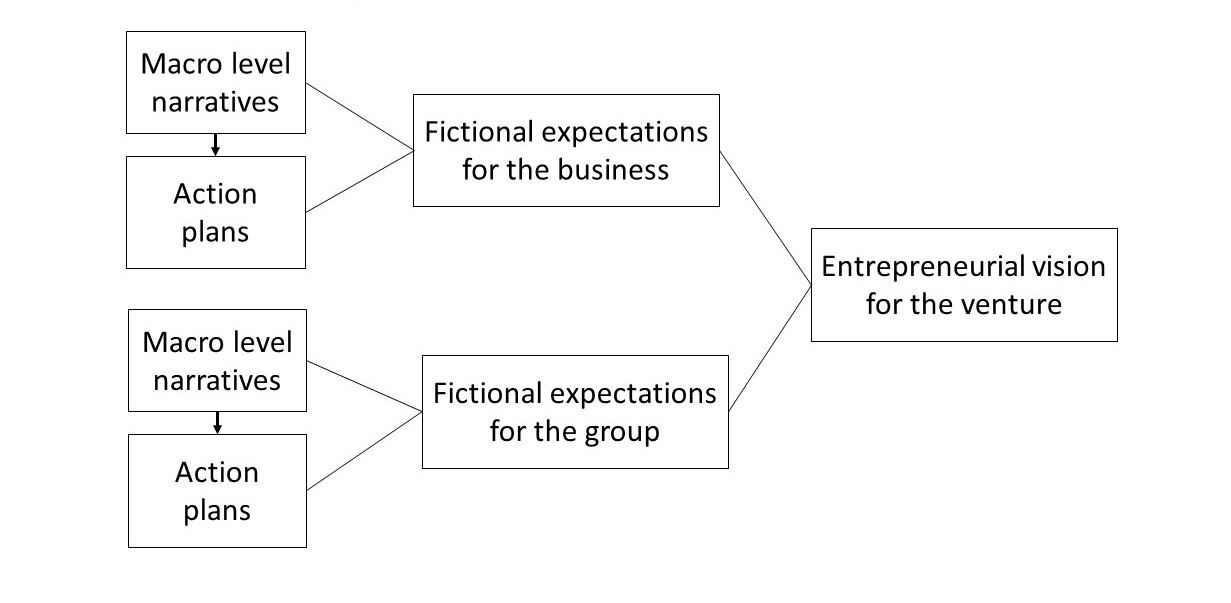
Table A3a: Code Structure Fictional Expectation for the Business


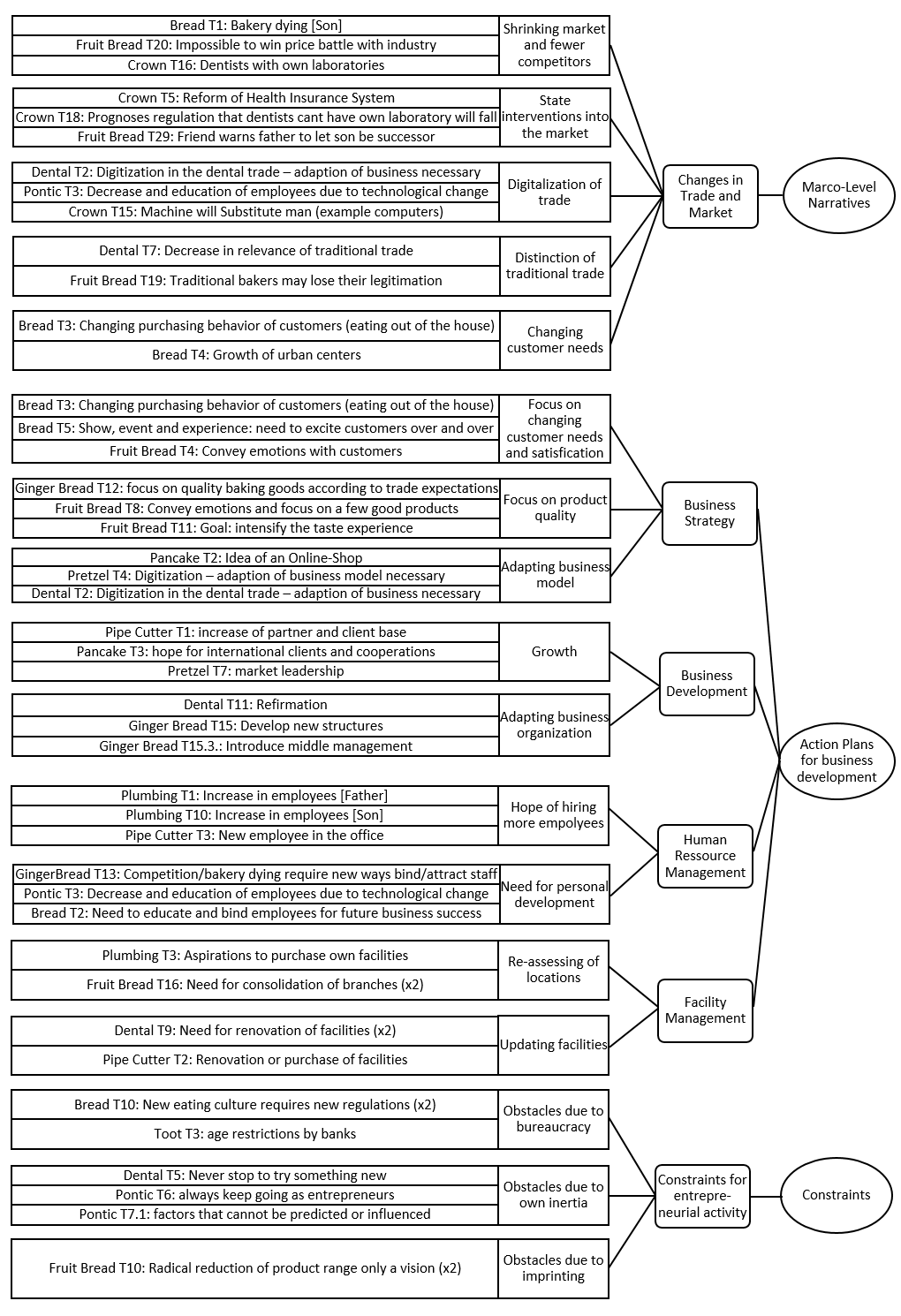


Table A3b: Code Structure Fictional Expectations for the Group


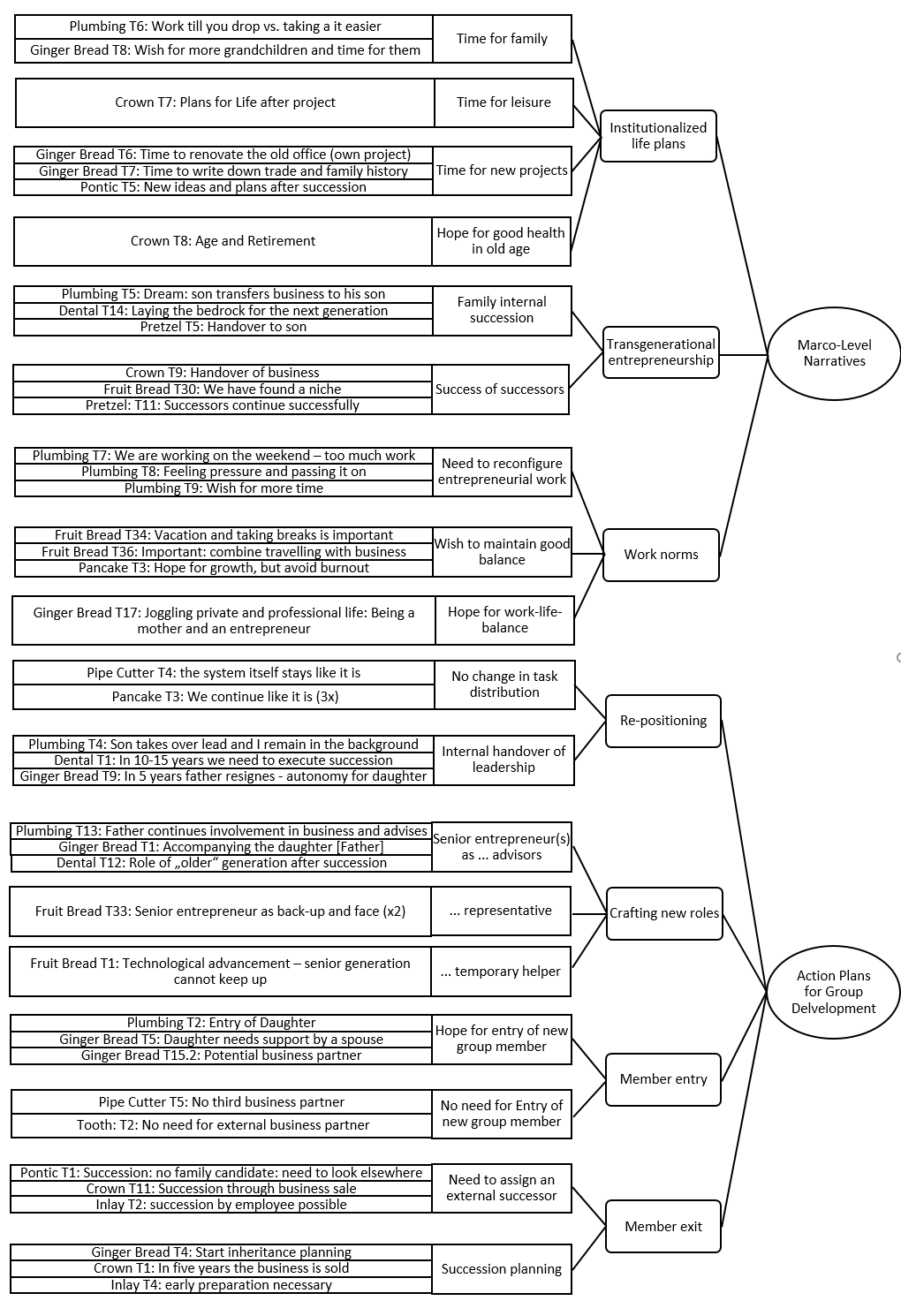


Table A4: Two Forms of Future Orientation as Content Element of entrepreneurial vision

| **Group** | **Future Orientation** | **Statements** |
| --- | --- | --- |
| Pipe Cutter | continuing | P2: Yes, we have our employees for now. The ones we have, we are very satisfied with. Uh, for sure we could have a few more employees who are reliable, uh, but otherwise we are in a good position at the moment, that we can more or less choose the orders, that we really don't have to fight for every order, but can do the orders we do conscientiously and calmly and uh, yes, actually also financially well [...] we are actually in a very good position. |
| Tooth | continuing | P1: „No but um so other rooms we don't want. P2: No. P1: we don't want other employees. P2: we don't want more employees. P1: we don't want more either, nope.  P2: "We continue to make the work processes more effective, but these are all so many new technologies in the 3D printing area and the CC area, so we don't necessarily need more technicians to make things, but we also need more technology to expand our sales, which has always worked out well so far.“ |
| Pontic | continuing | P2: and you [as an entrepreneur] actually only ever move when you're not doing well anymore that's also such a human characteristic say now the pressure is on here that we somehow can't pay anything anymore or can't pay our employees anymore I hope it never gets to that point." |
| Bread | continuing | P2: I see for the future a very [...] so I see that the companies that are successful have managed to be successful because they have done their homework well in the personnel area, that is, companies that manage to qualify their employees and to bind them to the company [...] it's not even the case that you now have to deal with how to grow particularly quickly, where do I see market opportunities or something, but the crux of the matter is personnel with proper know-how and if we succeed in binding our employees [...], we will be just as successful in 10-20 years as we are now.  P2: So we have come to a relative agreement that this thing [construction of the adventure farm] can work excellently at this location, but we would need. P1: 100 people P2: 100 good employees and at least 10 of them excellent managers, and we don't see that we can win them over at the moment. |
| Pretzel | divergent | P4: "At the end of the day, if you look at McDonalds, the new McDonalds branches, where you can also order and pay by app, where you order in the store at the order terminal and have it delivered to your table, these are the topics that we are dealing with, I'll say. P1: we see. P4: us where we uhm in different thoughts and projects on it. P1: yes. P4: work towards it yes and until then our business will certainly change yes". |
| Fruit Bread | divergent | P1 : and in my opinion, we will perhaps add to this by specializing even more, by reducing the products, and by closing some locations  P3 (employee/son): "and maybe in the future there will be some changes in the ways or in the processes and so on and we will definitely change things and um yes like everywhere in today's world you just try to use the space in the best possible way and that will definitely play a role for us in the future um yes." |
| Ginger Bread | divergent | P2: "I know that I want to produce good baked goods in [a town in Bavaria] and that the quality of the craftsmanship is really important to me and that we still have a long way to go because my grandfather built a large bakery where the goal was to produce as many rye breads and light rolls as possible and that in the meantime, first of all, that's not what I want to do and secondly, the competition (laughs) is far too great. |
| Dental | divergent | P3: „"As I said, there is a certain change in the way of working and the workflow and I think that in the next ten to fifteen years there will be a lot of development, especially with 3D printing technology and all that, and that in principle we will also develop in such a way that we will go more into this digital treatment [...] there are certain plans that one might [...] perhaps bring more practitioners into the house in order to save ways to work more effectively and perhaps develop new concepts."  P2: Yes, but (P3) always has ideas, he also collects these things, because he is really very innovative and thinks very, very modern. And of course, he comes up with things that make you gulp a bit at first and then think about it: Oh, that's not really so wrong. We'll have to think about what we can do with it, won't we? |

Table A5: Statements on Role Confidence (Condition 1)

| Summary of role expectations | | Quotes | Tonality |
| --- | --- | --- | --- |
| *Clarity and high certainty of own and mutual expectations* | |  |  |
| Pipe Cutter | - Clear division of tasks and responsibilities - Agreement on behavioral expectations | 1 (owner-manager): Yes, otherwise, I'd say so, so the way it's going right now, we've actually had a system for years, where, I'd say, everyone has their, yes, I'd say, their hobbyhorse, which we then just want to expand. So we don't want to fundamentally change anything again. | familiar, open |
| Bread | - Clear division of tasks and responsibilities - Agreement on behavioral expectations | 1 (owner-manager/father): So the [retirement] is postponed for another 10 years I think.  2 (owner-manager/son): I'm not against it (laughs), that it's postponed (3) that's fine. | familiar, routinized |
| Tooth | - Clear timing for exit of older owner-manager - Agreement on behavioral expectations | 1 (owner-manager): I'm uh then [in 5 years] just going to retire. 2 (owner-manager): °just?° 1:yes or am already retired. 2: for a long time or (laughter). 1: for a long time. 2: (laughs). 1: (laughs) am then already 5 years then retired. 2: laughs. 1: no, but I'm going to retire in 5 years, so I'll only be doing some consulting. 2: which I also gladly accept of course. | familiar, joking |
| Pontic | - Clear division of tasks and responsibilities - Agreement on behavioral expectations | 2 (manager): So we are also in agreement in our entrepreneurial attitude: [...] So, if one, I'm simply exaggerating now, is a sleeping pill and the other is a complete runaway, then it's just difficult for it to work. Yes, so the mentality should already go a bit in the same direction, at least, yes. | familiar, joking |
| Dental | - Clear division of tasks and responsibilities - Agreement on behavioral expectations and future continuation of business | 3 (owner-manager/son): The older generation will never retire completely. 1 (owner-manager/mother): No. 3: So that's a fact they will leave on paper but they will. 1: yes. 3: the older generation will also let the others do their thing I suppose [...] they will then rather go over into the uh advisory activity thus that 1: yes [...]I would like to sit here evenly no more with 80 [years of age] and then perhaps uh 3: rattle around exactly here thus 1: Yes rattle around and occupy the junior uh job. | familiar, open |
| Ginger Bread | - Already informal handover of responsibility ("new boss") - Agreement on behavioral expectations (e.g. withdrawal of the current owner, assumption of responsibility by the future manager) | 2 (manager/daughter): And that my father is already relatively far with thinking about how he will continue in his future and has exactly a plan and says I also do not want to work forever that was already when I started five years ago six years ago so that he was clear that from the beginning it was clear that he won’t be doing this forever [...]. | familiar, open, caring |
| Fruit Bread | - Clear timing for takeover - Shared leadership and decision-making, taking into account future distribution of positions - Agreement on behavioral expectations (e.g., get involved - give space; retreat and substitute for vacation) | 1 (owner-manager/father): I will hand over responsibility for the company before my sixtieth birthday. Deadline but he has known that for a long time. 3 (employee/Son): Yes. 1: There comes the point where also 3: before your sixtieth? 1: Yes yes deadline. 3: I'll sign it right away. 1: Yes yes I’d say so but when exactly before my sixtieth… 3: with 59, great (laugh).  1: No, the situation is um how many companies are there where the old man doesn't give up the helm and in our family it is traditional already the third generation and with him it will be the fourth who will get the business relatively early[...] when I see how committed he is now and what all should be changed there um who have the opportunity to redesign it that way. | familiar,  joking,  routinized |
| Prezel | - Clear division of tasks and responsibilities - Agreement on behavioral expectations - Awareness of respective position in the company (special role of external manager, minority shareholder, face of the family business) | 4 (manager): I don't want to butter anyone up [...] but what strikes me is that craft businesses in particular often have the issue [...] that the founders or the operators don't let go and remain active in the company in a patriarchal way [...] until they are carried out in a tin coffin and the successor generation never really has the opportunity to take on responsibility [...] yes, and I noticed that the family Paula Brezel and Berthold Brezel did a very exemplary job [...] of giving them responsibility very early on [...]. Yes, and I have noticed that the family Paula Brezel and Berthold Brezel have done a very exemplary job [...] have given responsibility to the children very early on and have also transferred shares in the company to them [...] and are now really letting go [...] and that is the basic prerequisite [...] that this can function at all, yes. | routinized, tensed between 1 and 2,  3 mediating, de-escalating, flattering |
| *Ambiguity and low certainty of own and each other's role expectations.* | |  |  |
| Pancake | - Currently clear distribution of tasks and responsibilities - But ambiguity on the part of the owner regarding the timing and general competence of the planned successor. - Wistfulness about alternative career plans of the daughter | Interviewer: When do you mean when you will hand this over to Max (=4) because now you meant ok this is always handed over to the son?" 1 (owner-manager/father): yes I have to tell you that's a good question that's one today that's yes you know that's puh that's uh uh [...] as a self-employed person you know that and and you also have to take a lot more responsibility and not that you can't do that you can do that too but it takes a certain discipline to do that a one and you have to have that first you know [...] I like to do my work I am happy about the bakery I would like to continue it for decades and also hand it over to Max and you have to have the feeling and the love for it and also uh uh then it will be a sure-fire success. | Exhaustive, evasive, waiting |
| Crown | - Currently clear distribution of tasks and responsibilities - But ambiguity on the part of the owner regarding timing and will/skill of potential successors | 1 (owner-manager): We'll have to wait and see for the next few years, she (employee/wife) has to prove herself first, whether she enjoys it at all, because you can be so experienced with money transactions [...] so either she has the talents to increase it or she says it's no fun. | familiar, evasive, waiting |
| Plumbing | - Currently clear distribution of tasks and responsibilities - But ambiguity of the owner regarding the way of his own future engagement in the company - Demand of "proving oneself" of planned successor | 1 (owner-manager/father): So since 2015 we or better I have been working on making him (3, deputy manager, son) broader and more stable [...]. So becoming a master craftsman is not difficult, but being a master craftsman is very [...] because if a young person does his master craftsman's diploma right after his training, he has a title but he cannot lead or manage and he also lacks professional experience. | familiar, joking |
| Oil | - Currently clear distribution of tasks and responsibilities - But ambiguity on the part of the owner regarding the timing and competence of the planned successor. - Demand of "proving oneself" of planned successor | 1 (owner-manager): There are also three people crystallizing out for the future management in ten years, maybe a bit earlier, including Jan (3, deputy manager, son), who will then have the management here [....], but who will then become the managing directors and authorized signatories will then become apparent in the next few years. | Demanding, self-proving, professionalized |

Table A6: Identifying Discourse Positions as Preparatory Step to Identify Hierarchical Congruence (Condition 2)

|  | **Leader** | **Follower** | **Newcomer** | **Partner** |
| --- | --- | --- | --- | --- |
| Right  to speak | - opens and/or ends conversation - distributes right to speak - has right to speak for and about - others; interrupts other(s) | - speaks mainly only after given right to speak - hardly/not at all actively intervenes from him or herself | - usually does not open the conversation, but can end it - only speaks after being given right to speak | - opens and/or ends conversation |
| Speech proportion | - high or low dominance of conversation, sets the topics - takes time to present own position and ideas - gives other(s) (more or less) time for own explanations | - degree of involvement varies - hesitant to self-positioning, but not assertive - aware of own role limitations in thinking and talking about future | - takes time - is present and ambitious, but not dominant - follows set topic, also brings own topics and positions in a clear, self-confident and proving way | - Takes and gives time to present own position and ideas for the future - no dominance - topics are set and talked about together |
| Reference  to other members | - Explanations from other(s) are supported and validated, but also improved, corrected and (de)evaluated, by using own explanations, background information, and referring to own/past achievements and experiences. - Shows a general appreciation of the other(s), but takes right to criticize and discredit others, which creates hierarchical distance. | - Hardly any reference to other members and their explanations | - Explanations of the other(s) are validated, but also openly or subtly criticized. Demarcation openly or in a subtle, well-dosed manner. Critical remarks by others are left uncommented. - Shows a general appreciation of the other(s) and the past, but also expresses wishes for possible changes and further developments. | - Explanation of other(s) are directly referred to, reinforced, validated and elaborated, using further examples, arguments. Also corrections and criticism are possible. - Shows appreciation for and deep knowledge about others, despite possible differences, a strong sense of belonging to other(s). |
| Way of talking about future | - Has a (long term) plan and clear ideas, which s/he voices and defends with determination and confidence | - No or only vague ideas, seems overstrained, uncertain and remains very vague and cautious in her/his statements | - Signals the willingness to shape the future by voicing clearly or hinting at own ideas and evaluations. | - Has ideas about future, which s/he voices and discusses |

Table A7: Deriving Narrative Hierarchies as Preparatory Step to Identify Hierarchical Congruence (Condition 2)

|  | **Neglected** | **Consecutive** | **Hybrid** | **Interlocked** | **Unisono** |
| --- | --- | --- | --- | --- | --- |
| Combination of Discourse Positions | Leader – Follower | Leader – Newcomer | Leader – Newcomer | Partners | Partners |
| Description | Unequally involved roles; clear dominance of leader, who represents the group in the interview and sets the topics and focus; follower is denied competence to talk about future issues | Unequally involved roles, dominance of leader, who primarily represents the group and sets topics; newcomer with competence and right to talk about future issues, follows topics and presents carefully and subtly own positions | Equally involved but unequal roles; phased dominance of leader, but also active involvement of newcomer. Newcomer with competence to talk about future issues, openly presents own positions | Equal roles at eye-level, all with competence and right to talk about future issues; depending on topic, slightly dominance of one partner possible | Equal roles at eye-level, all with competence and right to talk about future issues; hardly any dominance of one partner |
| Discourse organization | No co-narrations, discussions or arguments; in extreme cases, no direct reference to follower; tensions or conflicts are not addressed, rather hidden | Separate, successively arranged blocks of speech, hardly co-narrations, discussions or direct mutual references | First separate arranged block of speech of leader, then active involvement of newcomer possible, with direct or reported discussions, and direct or indirectly mutual references | High degree of mutual direct references, usually validating and supporting; depending on topic (unisono) co-narrations but also open disagreements and discussions | High degree of mutual supporting and validating direct references, a lot of co-narrations and partly unisono speaking |
| Collective Identity | „I“ (leader) and „you“ (follower), no „we“ | „We“, with emphasis on „I“ and „you“; Leader as instructor with goal to groom newcomer | Solidary „we“, common goal to hand/take over | Trusting and strong „we“, appreciation and awareness of differences | Trusting and strong „we“, strong sense of unity |
| Cases | Crown  Pancake | Oil  Plumbing | Ginger Bread  Fruit Bread  Dental | Bread  Pontic  Pretzel | Pipecutter  Tooth |
| Hierarchy | asymmetric | asymmetric | asymmetric / symmetric | symmetric | symmetric |
